# Influenza A Virus Nucleoprotein Activates the JNK Stress-Signaling Pathway for Viral Replication by Sequestering Host Filamin A Protein

**DOI:** 10.3389/fmicb.2020.581867

**Published:** 2020-09-25

**Authors:** Anshika Sharma, Jyoti Batra, Olga Stuchlik, Matthew S. Reed, Jan Pohl, Vincent T. K. Chow, Suryaprakash Sambhara, Sunil K. Lal

**Affiliations:** ^1^School of Science, Monash University Malaysia, Subang Jaya, Malaysia; ^2^National Center for Emerging Zoonotic and Infectious Diseases, Centers for Disease Control and Prevention, Atlanta, GA, United States; ^3^Department of Microbiology and Immunology, Yong Loo Lin School of Medicine, National University of Singapore, Singapore, Singapore; ^4^Influenza Division, National Center for Immunization and Respiratory Diseases, Centers for Disease Control and Prevention, Atlanta, GA, United States; ^5^Tropical Medicine and Biology Multidisciplinary Platform, Monash University Malaysia, Subang Jaya, Malaysia

**Keywords:** protein-protein interaction, host-virus interaction, actin-binding proteins, IAV replication, next generation anti-influenza target

## Abstract

Influenza A virus (IAV) poses a major threat to global public health and is known to employ various strategies to usurp the host machinery for survival. Due to its fast-evolving nature, IAVs tend to escape the effect of available drugs and vaccines thus, prompting the development of novel antiviral strategies. High-throughput mass spectrometric screen of host-IAV interacting partners revealed host Filamin A (FLNA), an actin-binding protein involved in regulating multiple signaling pathways, as an interaction partner of IAV nucleoprotein (NP). In this study, we found that the IAV NP interrupts host FLNA-TRAF2 interaction by interacting with FLNA thus, resulting in increased levels of free, displaced TRAF2 molecules available for TRAF2-ASK1 mediated JNK pathway activation, a pathway critical to maintaining efficient viral replication. In addition, siRNA-mediated FLNA silencing was found to promote IAV replication (87% increase) while FLNA-overexpression impaired IAV replication (65% decrease). IAV NP was observed to be a crucial viral factor required to attain FLNA mRNA and protein attenuation post-IAV infection for efficient viral replication. Our results reveal FLNA to be a host factor with antiviral potential hitherto unknown to be involved in the IAV replication cycle thus, opening new possibilities of FLNA-NP interaction as a candidate anti-influenza drug development target.

## Introduction

Influenza A viruses (IAVs) are the most popular amongst the Influenza viruses, owing to their ability to cause epidemics and pandemics (Taubenberger and Morens, 2009). In humans, infection with contemporary IAV strains can proceed without symptoms or may be accompanied by fever, headaches, fatigue, chills, sore throat, and rhinorrhea (nasal discharge) (Soleimani and Akbarpour, 2011). The elderly and individuals with weakened immune system or chronic health problems may develop serious and potentially life-threatening medical complications, such as bronchitis and/or pneumonia, post-IAV infection (Falsey and Walsh, 2006; Liu et al., 2016). The virulence of IAV is due to its easy spread via aerosols and the frequent changes in the viral surface antigen through antigenic drift and antigenic shift, which enables it to escape from the host’s immune system (Shimizu, 2000; Diaz et al., 2013).

Viruses, being obligate parasites, are dependent on the living host cell for their replication (Samji, 2009). The discovery of host factors necessary for virus replication opened broad possibilities for the development of next generation antiviral drugs (Thorne et al., 2016). Instead of targeting viral proteins, this novel pharmacological strategy aims to target essential host proteins and pathways used by the IAVs to fulfill their replicative cycle (Qian et al., 2016). Therefore, this strategy also aids in overcoming the serious challenge of viruses escaping from drug effects via antigenic drift or shift (Linero et al., 2012). One of the major viral proteins known to play a critical role during IAV replication is nucleoprotein (NP). According to Li et al. (2015), the IAV NP has been reported to interact with both host and viral proteins to mediate the transport of the vRNP from the cytoplasm to the nucleus and vice versa, as well as to regulate the expression of vRNA. The sequence of NP has shown to be conserved across IAV isolates thus, NP-host interactions serve as compelling antiviral targets (Sharma et al., 2014).

Preliminary data from our lab obtained via high-throughput mass spectrometric analysis of IAV NP co-immunoprecipitation protein pull-down showed putative interaction between host Filamin A (FLNA) protein and IAV NP. FLNA belongs to the filamin protein family, which consist of three members, Filamin A, B, and C, of which FLNA is known to be the most abundantly expressed and widely distributed (Wang et al., 2015). By cross-linking actin filaments, FLNA can finely tune the dynamic three-dimensional structure of a cell. In addition, FLNA has been reported to interact with more than 90 functionally diverse range of cellular proteins, including cytosolic effector proteins, adhesion proteins, transmembrane proteins, DNA damage repair and transcription proteins, signaling molecules, and ion channel proteins (Yue et al., 2013; Wang et al., 2016). Because of the diverse range of interacting partners, FLNA plays a role in integrating cellular mechanics as well as acts as a signaling scaffold by connecting cellular processes to the dynamic regulation of the actin cytoskeleton (Stossel et al., 2001; Feng and Walsh, 2004). FLNA has been reported to primarily be a cytoplasmic protein, however, recent studies have reported localization of FLNA in the nucleolus thus, making it an interesting target of study, considering that IAV replication occurs in the nucleus (Deng et al., 2012).

In general, FLNA has been reported to play a key role in the establishment and replication of a diverse range of viral pathogens (Malathi et al., 2014). According to Jiménez-Baranda et al. (2007), FLNA acts as an adaptor protein that links the HIV-1 virus receptors to the actin cytoskeleton to promote virus entry. In addition, Cooper et al. (2011) reported interaction between HIV-1 Gag protein and FLNA for the subsequent intracellular trafficking of viral proteins, leading to efficient virus replication, assembly, and release. FLNA has also been documented as an integral protein required for the life cycle of Hepatitis C virus, adenoviruses, and coxsackievirus (Shafren et al., 1995; Kälin et al., 2010; Ghosh et al., 2011). More interestingly, respiratory syncytial virus, a negative sense RNA virus like IAV, has also been reported to co-localize with FLNA although the significance of the interaction has yet to be established (Shaikh et al., 2012).

The interaction between FLNA and IAV NP and the role of FLNA and its associated proteins in IAV replication yet remains to be elucidated. To this end, this study focuses on further unraveling the biochemical changes in IAV infected cells and the role of FLNA in the IAV replication cycle.

## Materials and Methods

### Cell Culture

Human lung adenocarcinoma (A549), human embryonic kidney 293 (HEK293), and Madin-Darby canine kidney (MDCK) cell lines (American Type Culture Collection, Manassas, VA, United States) were grown in an 5% CO_2_-containing environment and maintained in Hyclone’s Dulbecco’s modified Eagle’s medium (DMEM) supplemented with 10% fetal bovine serum (Hyclone, UT, United States) and penicillin–streptomycin solution (100 units per ml; Invitrogen, NY, United States).

### siRNA, Plasmids, and Antibodies

The FLNA siRNA pool was purchased from Santa Cruz Biotechnology (SCB) (TX, United States), NP siRNA pool was purchased from Sigma-Aldrich (MO, United States) and the Silencer^®^ Negative Control No. 1 siRNA was purchased from Applied Biosystems (CA, United States). The NP gene of A/Puerto Rico/8/34 (H1N1) (*PR8*), A/Hong Kong/1/1968 (H3N2) (*HK*), and A/WSN/1933 (H1N1) (*WSN*) were cloned into the pcDNA3.1 myc-vector (Invitrogen, NY, United States). The pEGFP-N1 plasmid was purchased from Addgene and pcDNA3-FLNA-GFP plasmid was kindly provided by the Calderwood Lab (Yale School of Medicine, CT, United States). The antibodies used in this study included anti-myc monoclonal antibody (mAB2276: Cell Signaling, MA, United States), anti-GAPDH monoclonal antibody (mAB2118: Cell Signaling), anti-vinculin monoclonal antibody (SC-73614: SCB), anti-GFP antibody (SC-9996: SCB), anti-FLNA monoclonal antibody (AB76289: Abcam, Cambridge, United Kingdom), anti-FLNA monoclonal antibody (SC-17749: SCB), anti-phospho-P53 monoclonal antibody (SC-377567: SCB), anti-P53 monoclonal antibody (SC-126: SCB), anti-caspase3 monoclonal antibody (SC-56053: SCB), anti-JNK1/2/3 monoclonal antibody (SC-7345: SCB), anti-phospho-JNK1/2/3 monoclonal antibody (SC-293136: SCB), anti-bax monoclonal antibody (SC-20067: SCB), anti-bad monoclonal antibody (SC-8044: SCB), anti-phospho-bad monoclonal antibody (SC-271963: SCB), anti-c-jun monoclonal antibody (SC-74543: SCB), anti-phosho-c-jun monoclonal antibody (SC-822: SCB), anti-ask1 monoclonal antibody (SC-5294: SCB), anti-phospho-ask1 monoclonal antibody (SC-166967: SCB), anti-traf2 monoclonal antibody (SC-365287: SCB), anti-traf2 polyclonal antibody (E-AB-60112: Elabscience, China), anti-NP monoclonal antibody (AB22285: Abcam), anti-NP monoclonal antibody (SC-101352: SCB), anti-NS1 monoclonal antibody (SC-130568: SCB), anti-M1 monoclonal antibody (SC-69824: SCB), Alexa Fluor^®^ 488 antibody (AB150073: Abcam), Alexa Fluor^®^ 594 antibody (AB150116: Abcam), antibody anti-mouse IgG HRP-linked antibody (#7076: Cell signaling), anti-rabbit IgG HRP-linked antibody (#7074: Cell Signaling), and mouse isotype control (#61656: Cell Signaling).

### Plasmid and siRNA Transfection

DNA transfections were carried out with Lipofectamine 2000 (Invitrogen) in serum-free DMEM medium. After 5 h of incubation, the serum-free medium was replaced with DMEM supplemented with 10% fetal bovine serum. siRNA transfections were performed similarly with Lipofectamine^®^ RNAiMAX Reagent (Thermo Fisher Scientific).

### Virus Infection and Plaque Assay

A/Puerto Rico/8/34 (H1N1) isolate and *HKX31* (H3N2), a recombinant virus that has the H3 and N2 segments derived from A/Aichi/2/1968 and all other proteins from A/Puerto Rico/8/34, strains of IAV were used for viral infection. Virus infection and plaque assays were performed as described in Batra et al. (2016).

### SDS-PAGE and Western Blotting

Cells were harvested in RIPA buffer (150 mM sodium chloride, 1% Triton-X, 50 mM Tris, pH 8.0, 10 mM EDTA, pH 13, 10 mM PMSF, PIC). Protein quantification was performed using the Protein Assay Bicinchoninate Kit (Nacalai Tesque, Japan) and 2 mg/ml BSA (Thermo Fisher Scientific) was used to construct the standard curve. The purified protein lysates were subjected to SDS-PAGE followed by Western Blotting. The blots were developed using the WesternBright enhanced chemiluminescent HRP substrate (Advansta, CA, United States) or Westar Supernova (Cyanagen, Italy) and the bands were captured with the ChemiDoc XRS imager (BioRad).

### Densitometry and Statistical Analysis

Western blot quantification/densitometry was performed using the ImageJ software. Statistical analysis was performed using the SPSS Software (IBM, IL, United States).

### Co-immunoprecipitation Assay

Cells washed with 1× PBS were resuspended in lysis buffer (150 mM sodium chloride, 0.5% NP-40, 20 mM Tris, pH 8.0, 10% glycerol, 2 mM EDTA, pH 13, 10 mM PMSF, PIC). Lysis was allowed to proceed at 4°C for 2 h. The homogenates were centrifuged at 10,000 rpm for 15 min. The supernatant was collected and incubated with the antibody against the targeted protein overnight at 4°C followed by 2 h incubation with Dynabeads Protein G (Thermo Fisher Scientific) (50 μl beads per sample – wash beads with PBS prior to use). The beads were washed with PBS thrice and the bound proteins were eluted by resuspension in 2X Laemmli buffer followed by 5–10 min of boiling. The eluates were subjected to SDS-PAGE and Western Blotting.

### MTT Assay

Adenocarcinomic human alveolar basal epithelial cells or HEK293 cells were cultured in a 96-well plate. MTT solution with a final concentration of 0.5 mg/ml (Sigma-Aldrich) was prepared in serum-free media (DMEM). Media from the 96-well plate was carefully aspirated and the cells were washed with 1× PBS once. 100 μl of the MTT solution was added into each well followed by incubation at 37°C, 5% CO_2_ for 3 h. After incubation, 150 μl of DMSO (Nacalai Tesque) was added into each well and incubated at 37°C, 5% CO_2_ for 30 min to completely dissolve the formazan crystals. The absorbance reading was then measured at OD = 570 nm and the % survival was calculated using the following formula: $\frac{A\,\left(t\,e\,s\,t\right)-A\,\left(b\,l\,a\,n\,k\right)}{A\,\left(c\,o\,n\,t\,r\,o\,l\right)-A\,\left(b\,l\,a\,n\,k\right)}$ ^∗^ 100.

### Immunofluorescence Microscopy Assay

Adenocarcinomic human alveolar basal epithelial cells or HEK293 cells were grown on a coverslip placed in a 24-well plate. The cells were fixed with 4% paraformaldehyde post-transfection or infection. 0.5% Trition-X was used to permeabilize the cells followed by blocking with 2% BSA solution. The cells were incubated with primary antibody targeting either FLNA or NP overnight followed by 2 h incubation in Alexa Fluor secondary antibody. The coverslip harboring the cells were mounted onto a glass slide using the ProLong^TM^ Gold Antifade Mountant with DAPI (Invitrogen). The images were captured using the Leica TCS SP5 II Confocal Imaging Microscope and analyzed using the Leica Application Suite X software.

### RNA Isolation, cDNA Conversion and qRT-PCR

RNA was isolated using the RNeasy^®^ kit (Qiagen, Germany) according to manufacturer’s protocol. 1 μg of the isolated RNA was converted into cDNA using the ReverTra Ace^®^ qPCR RT Master Mix with gDNA Remover kit (Toyobo, Japan). Quantitative real-time PCR was performed using the SensiFAST SYBR Hi-Rox Kit (Bioline, London, United Kingdom). The primers used are listed in Table 1. The amplification was performed using one cycle of 50°C for 20 s, one cycle of 95°C for 10 min, 40 cycles of 95°C for 15 s and respective annealing temperature for 1 min and followed by melt curve analysis. Quantitative real-time PCR was conducted using the 7500 Fast Real-Time PCR System (Applied Biosystems). The data was analyzed using the delta-delta-ct method.

**TABLE 1:** Primer sequence for qRT-PCR.

Primer name	Primer sequence(5′-3′)	References
NP_Forward	TGTGTATGGACCTGCCGTAGC	He et al., 2013
NP_Reverse	CCATCCACACCAGTTGACTCTTG	
FLNA_Forward	CATCAAGTACGGTGGTGACG	Mayanagi et al., 2008
FLNA_Reverse	ACATCCACCTCTGAGCCATC	
β-ACTIN_Forward	GAACGGTGAAGGTGACAG	
β -ACTIN_Reverse	TTTAGGATGGCAAGGGACT	
GAPDH_Forward	ACCCACTCCTCCACCTTTGAC	
GAPDH_Reverse	TCCACCACCCTGTTGCTGTAG	

## Results

### IAV NP Interacts With Human FLNA Protein in a Conserved Manner

To identify the human interacting partners of IAV NP (*PR8*), IAV PR8 infected A549 cells were subjected to co-immunoprecipitation (Co-IP) assay using anti-NP antibody. The eluted antibody-protein-protein complex was subjected to mass spectrometric analysis. Interaction between host Filamin A protein and IAV NP was observed in the nuclear and cytoplasmic fractions at 4 h.p.i and in the cytoplasmic fraction at 8 h.p.i (results not shown).

**FIGURE 1:**
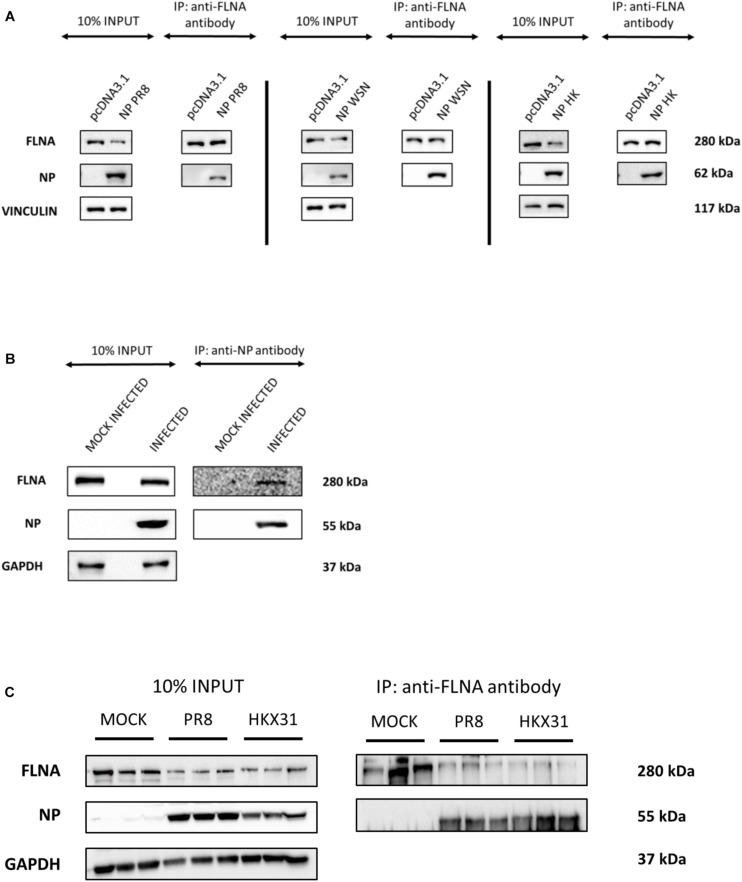
NP from the IAV interacts with host FLNA protein conservatively. **(A)** HEK293 cells were transfected with either 5 μg of pcDNA3.1, NP(*WSN*), NP(*HK*) or NP(*PR8*) plasmids (*n* = 3) (see Supplementary Figures 1–3). 24 h post-transfection, cells were harvested. IP was setup using mouse anti-FLNA antibody and NP was detected in the eluate by Western blotting using rabbit anti-NP antibody. **(B)** A549 cells were infected with IAV *PR8* (MOI = 1). 24 h post-infection, cells were harvested. IP was setup using mouse anti-NP antibody and FLNA was detected in the eluate by Western blotting using rabbit anti-FLNA antibody. **(C)** A549 cells were infected with IAV *PR8* (MOI = 5) and *HKX31* (MOI = 1) (*n* = 3). IP was setup using mouse anti-FLNA antibody and anti-IgG mouse antibody and NP was detected in the anti-FLNA antibody IP eluate by Western blotting using mouse anti-NP antibody (see Supplementary Figure 16).

To further validate the IAV NP-human FLNA interaction, Co-IP assay followed by Western blotting was performed. HEK293 cells were transfected with NP from the *PR8*, *HK* and Strain A/WSN/1933(H1N1) (*WSN*) isolates. The cells were harvested at 24 h post-transfection and subsequently reverse IPs were performed using anti-FLNA antibody. The results revealed a conservative interaction between host FLNA and IAV NP, with positive FLNA-NP interaction observed across NP from the *PR8*, *HK*, and *WSN* strains (Figure 1A). Subsequently, to confirm IAV NP-human FLNA interaction in an IAV microenvironment, A549 cells were infected with IAV *PR8* (MOI = 5) and *HKX31* (MOI = 1) followed by IP using either anti-NP or anti-FLNA antibody. The IAV NP of *PR8* was found to interact with human FLNA (Figure 1B) and reciprocally, FLNA was found to interact with NP from both *PR8* and *HKX31* strains (Figure 1C). Collectively, these results suggest a positive and conserved IAV NP-human FLNA interaction.

### IAV NP and Human FLNA Co-localize

**FIGURE 2:**
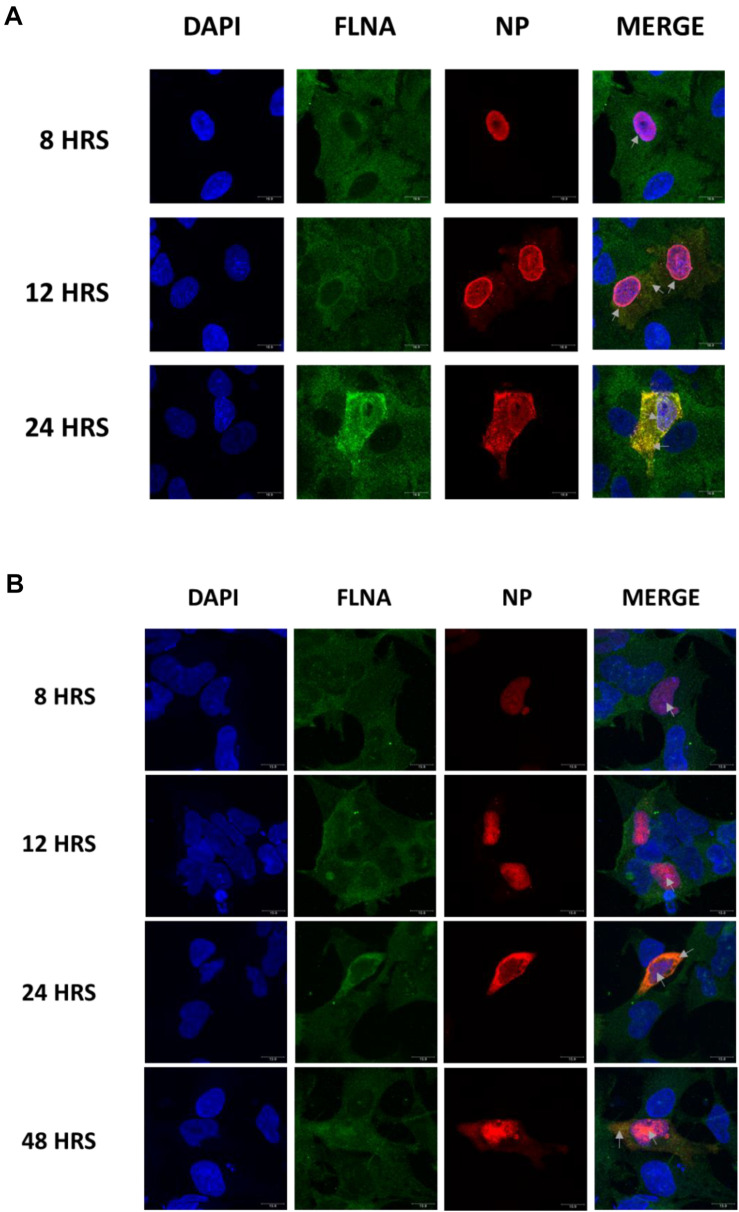
Host FLNA co-localizes with IAV *PR8* NP. **(A)** Shown is the cellular distribution of FLNA and NP proteins in IAV *PR8* infected (MOI = 1) A549 cells at respective timepoints (220.5× magnification). **(B)** Shown is the cellular distribution of FLNA and NP proteins in NP *PR8*-transfected HEK293 cells (3 μg plasmid transfection) at respective timepoints (220.5× magnification). NP and FLNA were detected using mouse anti-NP antibody and rabbit anti-FLNA antibody, respectively. Alexa flour 488 (green-FLNA) and 594 (red-NP) conjugated secondary antibody were used. ProLong Gold Antifade Mountant with DAPI was used to stain the nucleus (blue) and mount the coverslip.

To further elucidate the co-localization site of IAV NP and human FLNA, A549 cells infected with IAV *PR8* (MOI = 1) were subjected to immunofluorescence analysis. The cells were fixed at respective time points and subjected to blocking, primary antibody incubation, secondary antibody incubation, and subsequent mounting. As shown in Figure 2A, in an IAV microenvironment, FLNA-NP co-localization was observed at all timepoints. At 8 h post-infection (h.p.i), co-localization was observed in the perinucleus and nucleus, where IAV replication takes place. As infection progressed, at 12 h.p.i, FLNA was found to co-localize with NP majorly at the peri-nuclear region, potentially suggesting the involvement of FLNA in the transport of NP from the nucleus to the cytoplasm. In addition, at 12 h.p.i, co-localization was observed in the cytoplasm in vesicle-like structures, further suggesting the role of FLNA in NP/virus particle transport. At 24 h.p.i, FLNA was found to co-localize with NP in all regions of the cell.

Similarly, co-localization of FLNA and NP was tested in NP *PR8*-transfected HEK293 cells. As shown in Figure 2B, FLNA-NP co-localization was observed at all timepoints tested. At 8 and 12 h post-transfection, co-localization was observed primarily in the nucleus. On the other hand, at 24 and 48 h post-transfection, co-localization was observed in both the cytoplasm and the nucleus. These results suggest that the initial site of interaction between IAV NP and human FLNA is in the nucleus of the host cell.

### Human FLNA mRNA and Protein Levels Attenuated in the Presence of IAV NP

**FIGURE 3:**
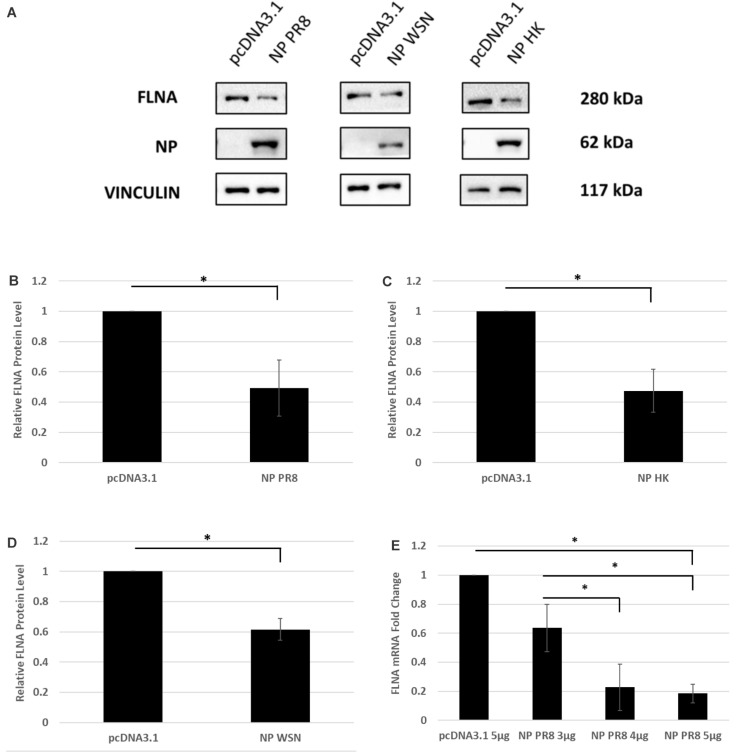
FLNA protein and mRNA levels attenuated in the presence of IAV NP. **(A)** HEK293 cells were transfected with 5 μg of either the pcDNA3.1 control plasmid or NP plasmid from the *PR8*, *HK*, or *WSN* strain. The cells were harvested with lysis buffer 24 h post-transfection and the purified protein lysate was subjected to SDS-PAGE and Western blot analysis. Blots were developed by ECL for FLNA, vinculin (loading control), and NP. Densitometric analysis was performed for **(B)** *PR8*, **(C)** *HK*, and **(D)** *WSN* transfected samples using the ImageJ software to visualize FLNA protein levels. The data show mean ± S.D. from one representative experiment (*n* = 3) (see Supplementary Figures 1–3). Statistical significance was determined using Student’s *t* test (^∗^*p* < 0.05). **(E)** HEK293 cells were transfected with pcDNA3.1 empty control plasmid or NP(*PR8*) plasmid in a dose-dependent manner. 24 h post-transfection, the cells were harvested, and total RNA was extracted followed by FLNA and GAPDH (loading control) mRNA estimation via qRT-PCR. Results are shown as mean ± S.D. of three independent experiments (*n* = 9). Statistical significance was determined using one-way ANOVA with *post-hoc* Tukey test (^∗^*p* < 0.05).

To determine the physiological implications of the NP-FLNA interaction, we aimed to study the effect of ectopic NP expression and IAV infection on FLNA levels. HEK293 cells were transfected with NP from the *PR8*, *HK*, and *WSN* strain and subsequently harvested at 24 h post-transfection for Western blot analysis against the FLNA protein. A significant decrease of approximately 2-folds in FLNA protein level was observed across the three NP strains tested (Figures 3A–D). Subsequently, to study the effect of NP on FLNA mRNA levels, HEK293 cells were transfected with the NP *PR8* plasmid in a dose-dependent manner and processed at 24 h post-transfection for RNA extraction and mRNA quantification. As seen in Figure 3E, a gradual decrease in FLNA mRNA levels was observed post-NP dose-dependent transfection, with approximately 5-folds attenuation of FLNA mRNA levels upon 5 μg of NP *PR8* plasmid transfection. These results suggest that the IAV NP plays a critical role in attaining FLNA attenuation and further emphasizes the importance of investigating the role of NP-FLNA interaction in the host cell and the implications of the interaction.

**FIGURE 4:**
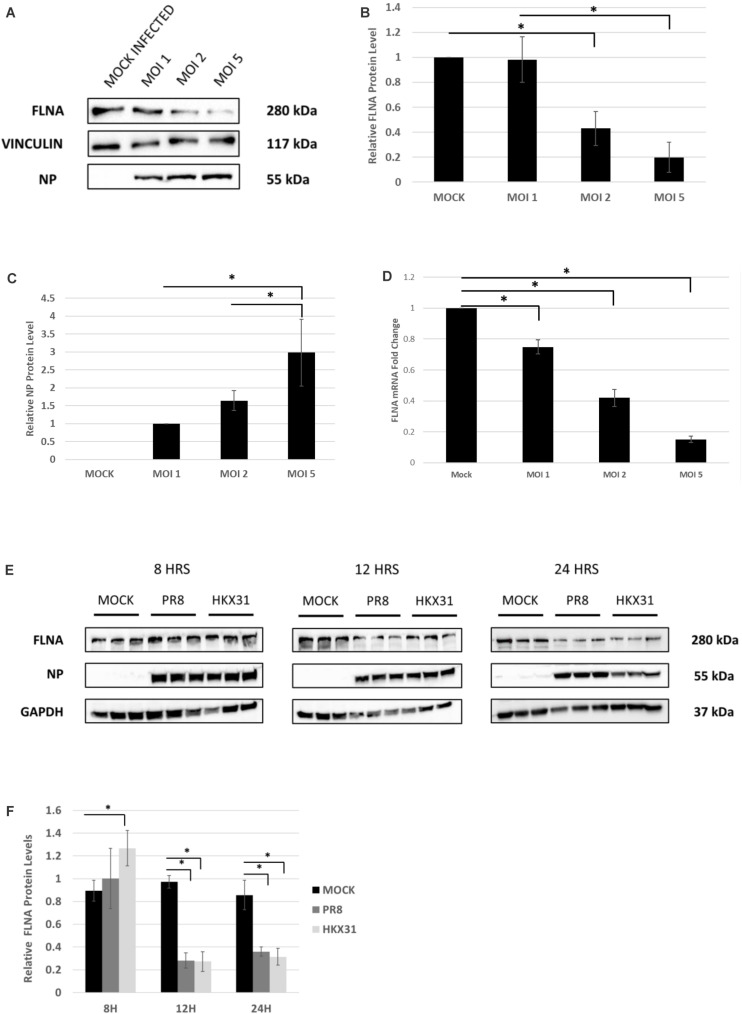
FLNA protein and mRNA levels attenuated in an IAV microenvironment. **(A)** A549 cells were infected with IAV *PR8* in a dose-dependent manner. 24 h.p.i the cells were harvested with RIPA buffer and 30 μg of protein lysate was loaded onto an SDS-PAGE gel followed by Western blotting. Blots were developed by ECL for FLNA, vinculin (loading control), and NP. **(B,C)** Densitometric analysis was performed for the dose-dependent IAV infected samples using the ImageJ software to visualize FLNA and NP protein levels. The data show mean ± S.D. from one representative experiment (*n* = 3) (see Supplementary Figure 5). Statistical significance was determined using one-way ANOVA with *post-hoc* Tukey test (^∗^*p* < 0.05). **(D)** A549 cells were infected with IAV *PR8* in a dose-dependent manner. 24 h.p.i the cells were harvested, and total RNA was extracted followed by FLNA and β-actin (loading control) mRNA estimation via qRT-PCR. Results are shown as mean of ±S.D. of three independent experiments (*n* = 9). Statistical significance was determined using one-way ANOVA with *post-hoc* Tukey test (^∗^*p* < 0.05). **(E)** A549 cells were infected with IAV *PR8* (MOI = 5) and *HKX31* (MOI = 1). The cells were harvested in a time-dependent manner with RIPA buffer and 30 μg of protein lysate was loaded onto an SDS-PAGE gel followed by Western blotting. Blots were developed by ECL for FLNA, GAPDH (loading control), and NP. **(F)** Densitometric analysis was performed for the infected samples harvested in a time-dependent manner using the ImageJ software to visualize FLNA protein levels. The data show mean ± S.D. from one representative experiment (*n* = 3). Statistical significance was determined using one-way ANOVA with *post-hoc* Tukey test (^∗^*p* < 0.05).

To examine the effect of whole virus infection on FLNA levels, A549 cells were infected with IAV *PR8* in a dose-dependent manner. 24 h.p.i, the cells were harvested and similarly processed to determine FLNA protein and mRNA levels. As seen in Figures 4A–D, a gradual decrease in FLNA protein and mRNA level was observed as the multiplicity of infection increased, with FLNA protein levels found to be significantly down-regulated by approximately 80% upon infection at MOI = 5 and FLNA mRNA levels found to be significantly down-regulated at MOI = 1, 2, and 5 by approximately 1. 3-, 2. 38-, and 6.7-folds, respectively.

Adenocarcinomic human alveolar basal epithelial cells cells were also infected with IAV *PR8* at MOI = 5 and *HKX31* at MOI = 1 and harvested in a time-dependent manner in order to study the effect on FLNA levels. Concordantly, FLNA protein levels were found to be down-regulated significantly at 12 and 24 h post-IAV *PR8* and *HKX31* infection (see Figures 4E,F). On the other hand, FLNA levels were found to be significantly upregulated in *HKX31* infected cells at 8 h.p.i. These results suggest that IAVs attenuate FLNA protein levels to its advantage, particularly in the later stages of IAV infection.

### IAV NP Is Essential to Attain Human FLNA Attenuation Post-infection

**FIGURE 5:**
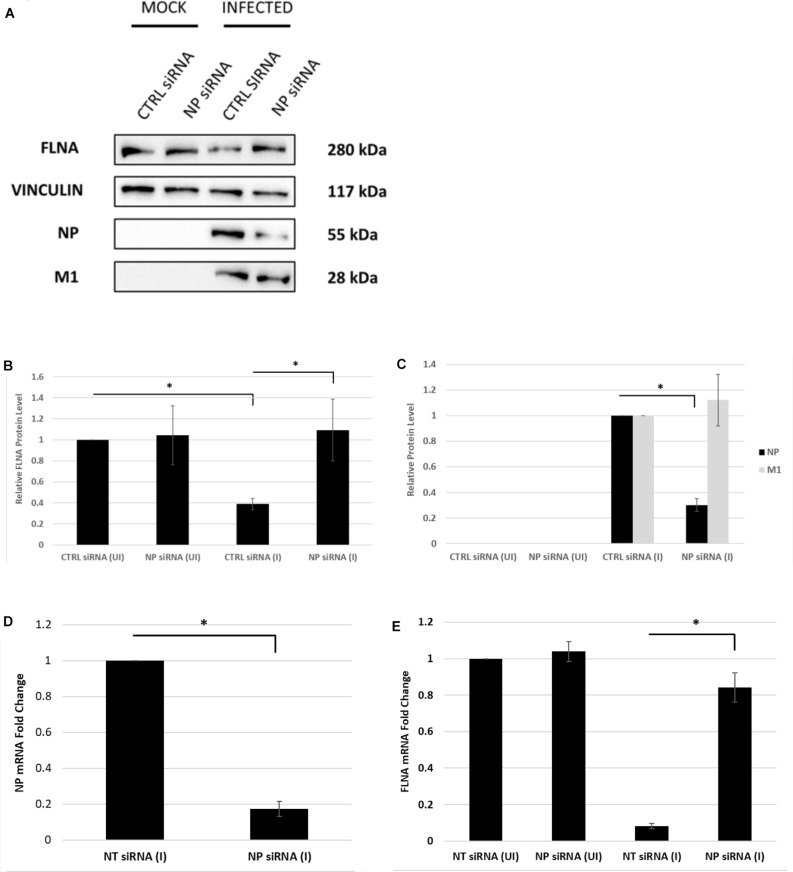
The NP of IAV is essential to attain FLNA attenuation post-IAV infection. **(A)** A549 cells were transfected with non-targeting control or NP siRNA (100 nM) for 24 h followed by IAV *PR8* infection (MOI = 5). 24 h.p.i, the cells were harvested with RIPA buffer and 30 μg of protein lysate was loaded onto an SDS-PAGE gel followed by Western blotting. Blots were developed by ECL for FLNA, NP, M1, and vinculin (loading control). **(B,C)** Densitometric analysis was performed using the ImageJ software to determine FLNA, M1, and NP relative protein levels. The data show mean ± S.D. from one representative experiment (*n* = 3) (see Supplementary Figure 6). Statistical significance was determined using one-way ANOVA with *post-hoc* Tukey test (^∗^*p* < 0.05). **(D,E)** A549 cells were transfected with non-targeting control or NP siRNA (100 nM) for 24 h followed by IAV *PR8* infection (MOI 5). The cells were harvested 24 h.p.i, and total RNA was extracted followed by NP, FLNA, GAPDH (loading control for NP), and β-actin (loading control for FLNA) mRNA estimation via qRT-PCR. Results are shown as mean ± S.D. of three independent experiments (*n* = 9). Statistical significance was determined using Student’s *t* test and one-way ANOVA with *post-hoc* Tukey test, respectively (^∗^*p* < 0.05).

Next, the effect of NP silencing on the levels of FLNA in an IAV microenvironment was studied. A549 cells were transfected with 100 nM pool of NP siRNA and then 24 h later infected with IAV *PR8* (MOI = 5). FLNA protein and mRNA levels were found to remain unchanged post-NP silencing (Figure 5). On the other hand, infected cells treated with non-targeting control siRNA showed approximately 60% reduction in FLNA protein levels and 12-folds downregulation in FLNA mRNA levels. It is important to note that NP silencing did not affect the viral transcription/translation processes as indicated by unchanged IAV M1 protein levels. Collectively, these results suggest that the presence of NP is necessary to attain FLNA attenuation post-IAV infection.

### Human FLNA Plays a Protective, Anti-viral Role Against IAV Replication

**FIGURE 6:**
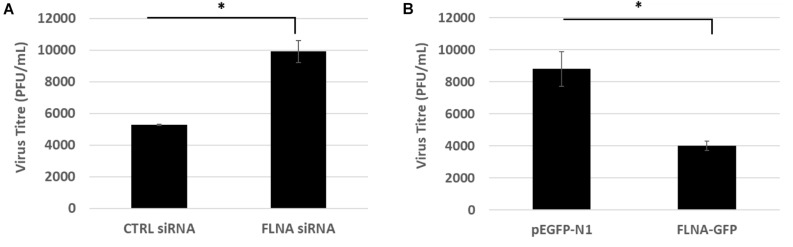
FLNA silencing significantly increases viral replication while FLNA over-expression significantly impairs viral replication. **(A)** Supernatant from siRNA treated and IAV *PR8* infected A549 cells were collected 24 h.p.i to perform plaque assay on MDCK cells. The data show mean ± S.D. from one representative FLNA silencing experiment (*n* = 3). Statistical significance was determined using Student’s *t* test (^∗^*p* < 0.05). **(B)** Supernatant from plasmid treated and IAV *PR8* infected HEK293 cells were collected 24 h.p.i to perform plaque assay on MDCK cells. The data show mean ± S.D. from one representative FLNA over-expression experiment (*n* = 3). Statistical significance was determined using Student’s *t* test (^∗^*p* < 0.05).

To study the effect of FLNA modulation on viral replication, A549 cells were transfected with FLNA siRNA and HEK293 cells were transfected with FLNA-GFP plasmid. The transfected cells were then infected with IAV *PR8* (MOI = 5). 24 h.p.i, the treated cells were harvested for protein (Western Blotting) and RNA extraction (qRT-PCR) and the supernatant, the media in which the cells were cultured in, was collected to perform plaque assay on MDCK cells. As seen in Figure 6, our plaque assay results indicated an 87% increase in virus titer post-endogenous FLNA silencing, thus suggesting an 87% increase in IAV replication rate. Concordantly, FLNA over-expression resulted in approximately 65% decrease in the IAV replication rate, further indicating the antiviral potential of FLNA (see Supplementary Tables 1, 2 for plaque counts).

**FIGURE 7:**
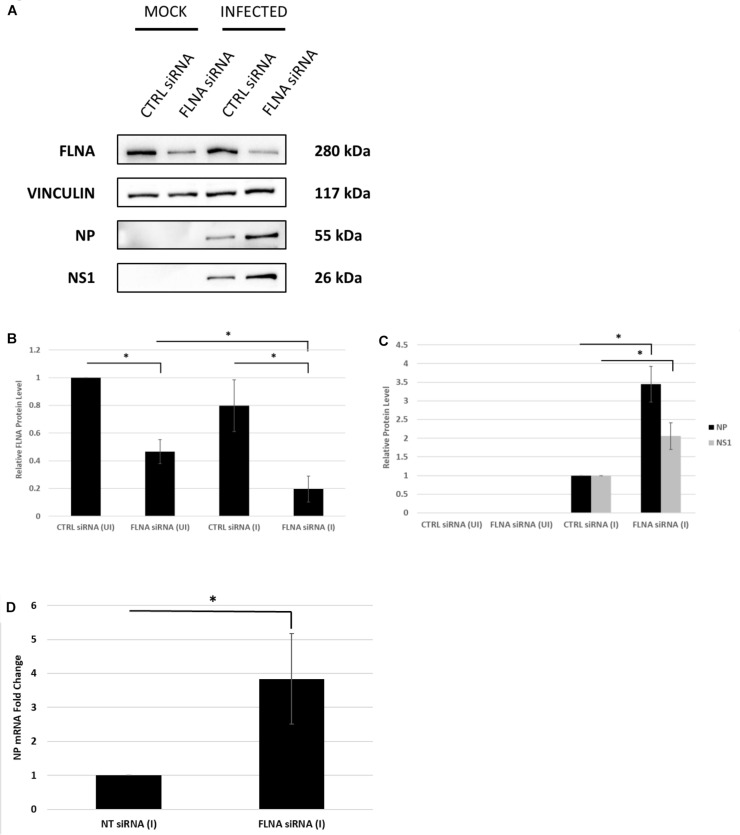
NP and NS1 protein levels and NP mRNA level significantly increased post-FLNA silencing in an IAV microenvironment. **(A)** A549 cells were transfected with FLNA (200 nM) or non-targeting control (CTRL) siRNA (200 nM) for 24 h followed by IAV *PR8* infection (MOI = 5). 24 h.p.i the cells were harvested with RIPA buffer and 30 μg of protein lysate was loaded onto an SDS-PAGE gel followed by Western blotting. Blots were developed by ECL for FLNA, vinculin (loading control), NP and NS1. **(B,C)** Densitometric analysis was performed for the infected, FLNA silenced samples using the ImageJ software to visualize FLNA and NP and NS1 protein levels. The data show mean ± S.D. from one representative experiment (*n* = 3) (see Supplementary Figure 7). Statistical significance was determined using one-way ANOVA with *post-hoc* Tukey test and Student’s *t* test, respectively (^∗^*p* < 0.05). **(D)** A549 cells were transfected with FLNA (200 nM) or non-targeting control (NT) siRNA (200 nM) for 24 h followed by IAV *PR8* infection (MOI = 5). 24 h.p.i the cells were harvested and total RNA was extracted followed by NP and β-actin (loading control) mRNA estimation via qRT-PCR. Results are shown as mean ± S.D. of three independent experiments (*n* = 9). Statistical significance was determined using Student’s *t* test (^∗^*p* < 0.05).

**FIGURE 8:**
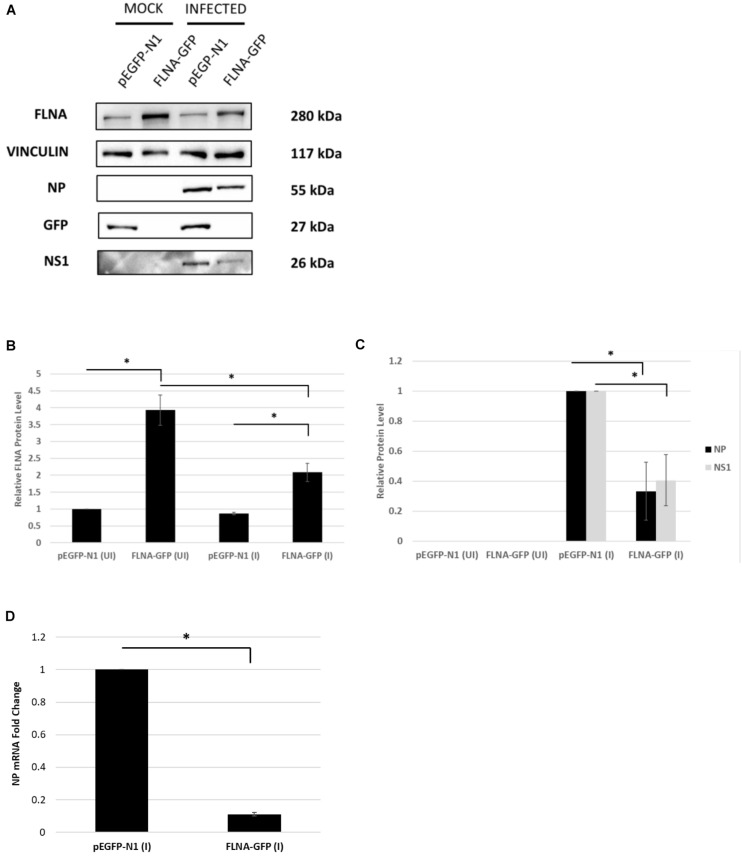
NP and NS1 protein levels and NP mRNA level significantly attenuated post-FLNA over-expression in an IAV microenvironment. **(A)** HEK293 cells were transfected with pEGFP-N1 or FLNA-GFP plasmid (3 μg) for 24 h followed by IAV *PR8* infection (MOI = 5). 24 h.p.i the cells were harvested with RIPA buffer and 30 μg of protein lysate was loaded onto an SDS-PAGE gel followed by Western blotting. Blots were developed by ECL for FLNA, vinculin (loading control), GFP, NP, and NS1. **(B,C)** Densitometric analysis was performed for the infected, FLNA over-expressed samples using the ImageJ software to visualize FLNA and NP and NS1 protein levels. Results are shown as mean ± S.D. of one independent experiment (*n* = 3) (see Supplementary Figure 9). Statistical significance for FLNA and NP was determined using one-way ANOVA with *post-hoc* Tukey test and Student’s *t* test, respectively (^∗^*p* < 0.05). **(D)** HEK293 cells were transfected with pEGFP-N1 or FLNA-GFP plasmid (3 μg) for 24 h followed by IAV *PR8* infection (MOI = 5). 24 h.p.i the cells were harvested and total RNA was extracted followed by NP and GAPDH (loading control) mRNA estimation via qRT-PCR. Results are shown as mean ± S.D. of three independent experiments (*n* = 9). Statistical significance for FLNA and NP was determined using Student’s *t* test (^∗^*p* < 0.05).

To further confirm changes in the IAV replication rate post-FLNA modulation, the treated cells were harvested to determine viral protein and mRNA levels. As shown in Figure 7, NP and NS1 protein levels and NP mRNA levels were found to be significantly higher post-FLNA silencing. On the other hand, NP and NS1 protein and NP mRNA levels were found to be significantly lower post FLNA-overexpression (Figure 8). These results are in concordance with the plaque assay results which revealed increased IAV replication post-FLNA silencing and attenuated IAV replication post-FLNA overexpression. Overall, it is suggested that FLNA plays a protective antiviral role in the host cell and in its absence, allows for better IAV replication.

### FLNA Prevents IAV-Induced JNK Activation and Apoptosis

**FIGURE 9:**
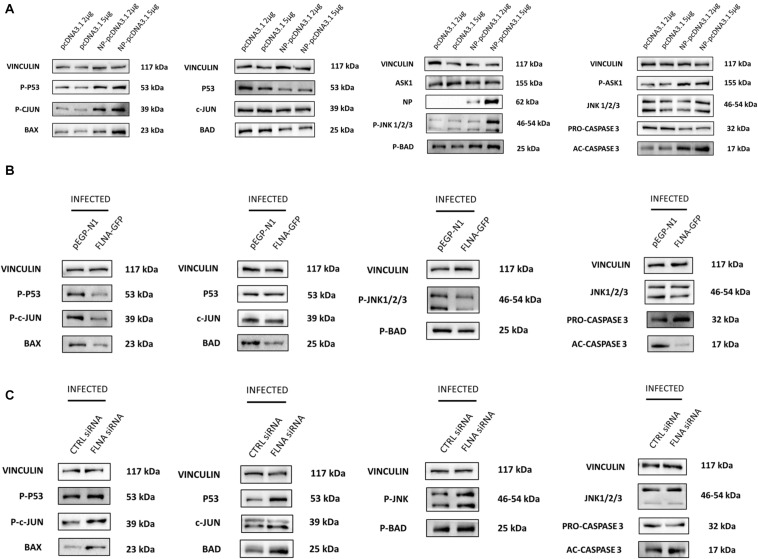
NP transfection and FLNA silencing in an IAV microenvironment were found to activate the JNK stress-signaling pathway while FLNA over-expression in an IAV microenvironment resulted in decreased JNK stress-signaling pathway activation. **(A)** HEK293 cells were transfected with either pcDNA3.1 empty control plasmid or NP *PR8* plasmid in a dose-dependent manner. The cells were harvested with RIPA buffer 24 h post-transfection and 30 μg of protein lysate was loaded onto an SDS-PAGE gel followed by Western blotting. Blots were developed by ECL for FLNA, vinculin (loading control), NP and JNK stress signaling pathway-associated markers. Representative blots are shown from one independent experiment (*n* = 3) (see Supplementary Figure 9). **(B)** HEK293 cells were transfected with either control pEGFP-N1 plasmid (3 μg) or FLNA-GFP plasmid (3 μg) followed by IAV *PR8* infection (MOI = 5) 24 h post-transfection. The cells were harvested with RIPA buffer 24 h.p.i and 30 μg of protein lysate was loaded onto an SDS-PAGE gel followed by Western blotting. Blots were developed by ECL for JNK stress signaling pathway-associated markers. Representative blots are shown from one independent experiment (*n* = 3) (see Supplementary Figure 10). See Figure 8 for confirmation of FLNA overexpression and IAV *PR8* infection. **(C)** A549 cells were transfected with either non-targeting control siRNA (200 nM) or FLNA siRNA (200 nM) followed by IAV *PR8* infection (MOI = 5) 24 h post-transfection. The cells were harvested with RIPA buffer 24 h.p.i and 30 μg of protein lysate was loaded onto an SDS-PAGE gel followed by Western blotting. Blots were developed by ECL for JNK stress signaling pathway-associated markers. Representative blots are shown from one independent experiment (*n* = 3) (see Supplementary Figure 11). See Figure 7 for confirmation of FLNA silencing and IAV *PR8* infection.

**FIGURE 10:**
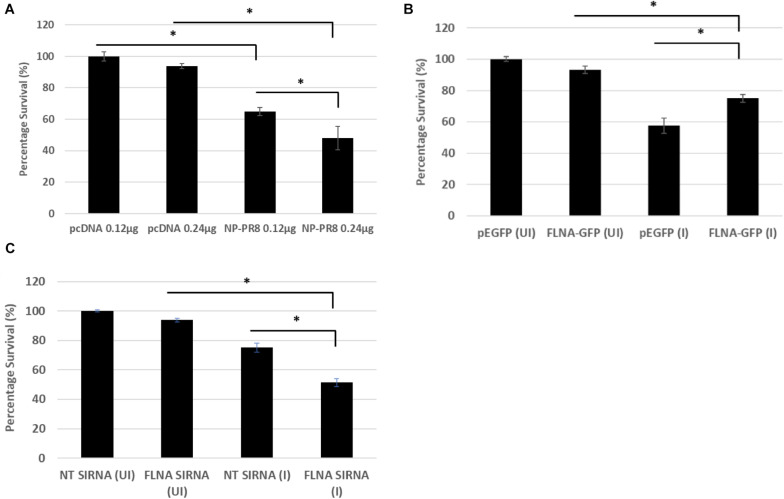
Reduced cell survival was observed post-NP transfection and FLNA silencing in an IAV microenvironment while increased cell survival was observed post-FLNA over-expression in an IAV microenvironment. **(A)** HEK293 cells were transfected with either pcDNA3.1 empty control vector or NP (*PR8*) plasmid in a dose-dependent manner. MTT assay was performed 24 h post-transfection. Results are shown as mean ± S.D. of one independent experiment (*n* = 5). Statistical significance was determined using one-way ANOVA with *post-hoc* Tukey test (^∗^*p* < 0.05). **(B)** HEK293 cells were transfected with pEGFP-N1 control plasmid (0.24 μg) or FLNA-GFP plasmid (0.24 μg) followed by IAV *PR8* infection (MOI = 5) 24 h post-transfection. MTT assay was performed 24 h.p.i. Results are shown as mean ± S.D. of one independent experiment (*n* = 5). Statistical significance was determined using one-way ANOVA with *post-hoc* Tukey test (^∗^*p* < 0.05). **(C)** A549 cells were transfected with non-targeting control siRNA (NT) (200 nM) or FLNA siRNA (200 nM) followed by IAV *PR8* infection (MOI = 5) 24 h post-transfection. MTT assay was performed 24 h.p.i. Results are shown as mean ± S.D. of one independent experiment (*n* = 5). Statistical significance was determined using one-way ANOVA with *post-hoc* Tukey test (^∗^*p* < 0.05).

Due to the involvement of FLNA in the stress-signaling pathway (Nomachi et al., 2008; Külshammer and Uhlirova, 2012; Yang et al., 2018), we were interested in further ascertaining the role of FLNA in the JNK stress-signaling pathway in the presence and absence of IAV NP. To this end, we first transfected HEK293 cells with NP(*PR8*) in a dose-dependent manner and subsequently processed the cells at 24 h post-transfection for quantification of the protein expression levels of JNK and its downstream effectors. Results indicated that at 24 h post-transfection, IAV NP induced JNK activation, as observed by increased JNK phosphorylation and activation of downstream cell death-related markers (Phospho-P53, activated-caspase 3, Phospho-c-Jun, Phospho-bad, and Bax) (Figure 9A). Concordantly, a dose-dependent decrease in cell viability was also observed 24 h post-NP transfection (Figure 10A). These results collectively suggest that NP is involved in initiating the required apoptotic responses in the host cell for efficient IAV replication.

Subsequently, to determine the role of FLNA in regulating the JNK signaling pathway during IAV infection, we either overexpressed or silenced FLNA in an IAV microenvironment and subsequently quantified the protein expression levels of JNK and its downstream effectors. Overexpression of FLNA resulted in reduced activation of the JNK stress-signaling pathway (lower phospho-JNK levels), decreased levels of JNK downstream cell death-related marker activation (Phospho-P53, activated-caspase 3, Phospho-c-Jun, Phospho-bad, and Bax) (Figure 9B), and increased cell viability (Figure 10B). Concordantly, FLNA silencing resulted in increased JNK stress-signaling pathway activation (higher phospho-JNK levels), increased levels of JNK downstream cell death-related marker activation (Phospho-P53, activated-caspase 3, Phospho-c-Jun, Phospho-bad, and Bax) (Figure 9C) and decreased cell viability (Figure 10C). The activation of the JNK stress-signaling pathway is a hallmark of IAV infection, thus, it is probable that the protective effect of FLNA against the IAV is through the suppression of the JNK stress-signaling pathway.

### IAV NP Interrupts FLNA-TNF Receptor-Associated Factor 2 (TRAF2) Interaction Leading to JNK Activation

**FIGURE 11:**
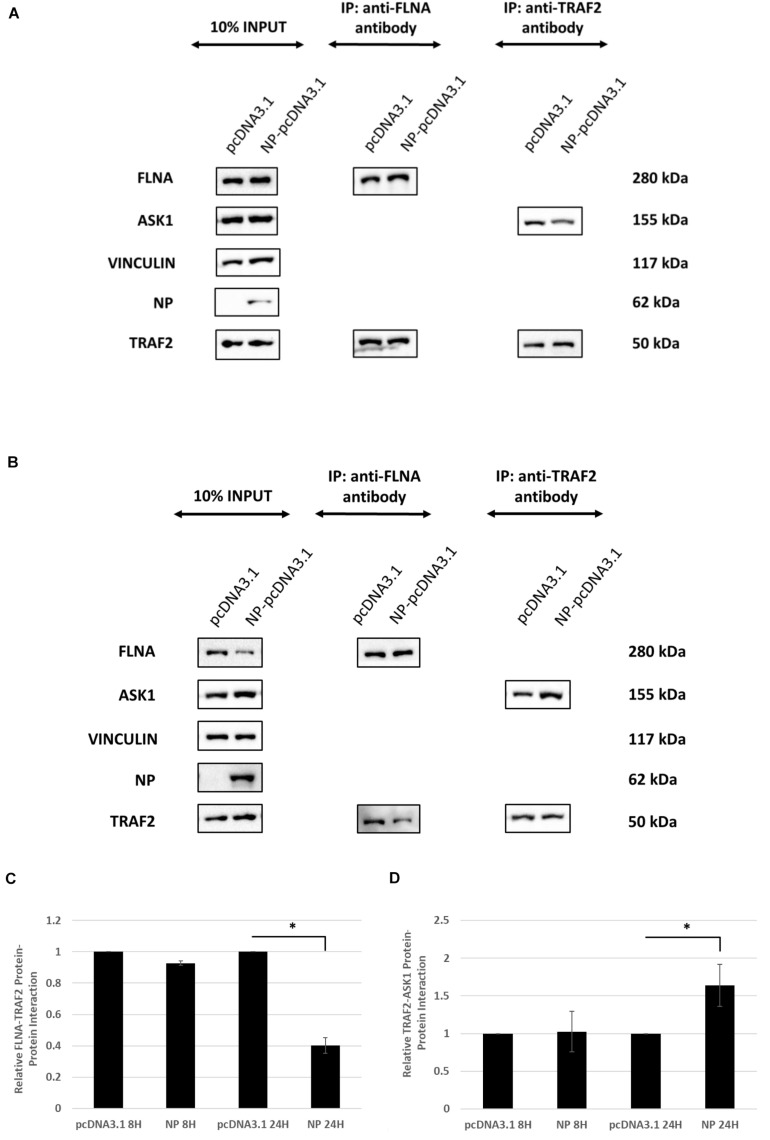
IAV NP interrupts FLNA-TRAF2 interaction, allowing for increased TRAF2-ASK1 interaction. HEK293 cells were transfected with either 5 μg of pcDNA3.1 empty control vector or NP(*PR8*) plasmid. The cells were harvested **(A)** 8 h post-transfection and **(B)** 24 h post-transfection and IP was setup using mouse anti-FLNA and mouse anti-TRAF2 antibody (*n* = 3) (see Supplementary Figures 1, 4). TRAF2 and ASK1 were detected in the eluate by Western blotting using rabbit anti-TRAF2 and mouse anti-ASK1 antibody. **(C,D)** Densitometric analysis was performed using the ImageJ software to visualize the level of FLNA-TRAF2 (FLNA used as loading control) and TRAF2-ASK1 (TRAF2 used as loading control) interaction. Results are shown as mean ± S.D. of one independent experiment (*n* = 3). Statistical significance for FLNA-TRAF2 and TRAF2-ASK1 level of interaction was determined using Student’s *t* test (^∗^*p* < 0.05).

**FIGURE 12:**
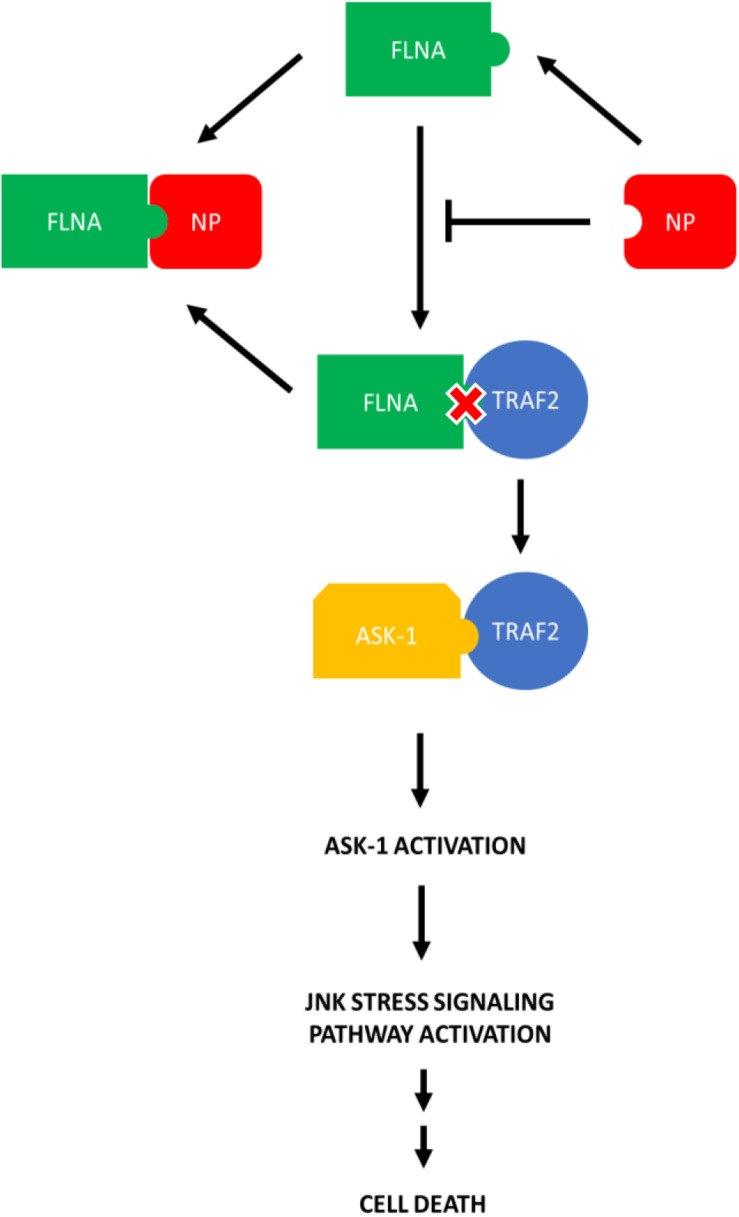
Model illustrating the process in which IAV NP interrupts FLNA-TRAF2 interaction thus allowing increased availability of TRAF2 for TRAF2-ASK-1 mediated JNK stress signaling pathway activation. The activation of the JNK stress signaling pathway resulted in an increase in the levels of apoptosis-related markers and hence, increased levels of cell death.

Since FLNA was found to play a crucial role in regulating the JNK stress-signaling pathway, we further examined the mechanism underlying FLNA-mediated JNK activation in the presence and absence of IAV NP. Through literature, FLNA is known to interact with TRAF2 (Leonardi et al., 2000; Campos et al., 2016), thus inhibiting TRAF2 from interacting with ASK1. TRAF2-ASK1 interaction has been reported to be crucial for JNK stress signaling pathway activation (Nishitoh et al., 1998). Through Co-IP, we observed a decrease in FLNA-TRAF2 interaction and an increase in TRAF2-ASK1 interaction at 24 h post-NP transfection (Figure 11). However, at 8 h post-NP transfection, the level of FLNA-TRAF2 and TRAF-ASK1 interactions were found to remain unchanged. The results suggest that the IAV NP interrupts the FLNA-TRAF2 interaction by sequestering FLNA and forming NP-FLNA interactions in the host cell at the later stages of IAV replication thus, allowing increased levels of free TRAF2 available for TRAF2-ASK1 interaction. As depicted in the model (Figure 12), increased levels of TRAF2-ASK1 interaction resulted in ASK1 activation, as seen by increased ASK1 phosphorylation (Figure 9A). The activation of ASK1, in turn, is known to activate the JNK stress-signaling pathway.

## Discussion

Filamin A has been reported to interact with more than 90 functionally diverse range of cellular proteins, including adhesion proteins, transmembrane receptors, transcription proteins, and signaling molecules, thus making it a key component of the host cell signaling scaffold (Savoy and Ghosh, 2013; Wang et al., 2015; Burns and Wang, 2017). In general, high-throughput virus-host interactomes revealed that viral proteins show preferential interaction with proteins having high number of direct interacting partners; making FLNA an interesting target of study (Dyer et al., 2008; Komarova et al., 2011; Munier et al., 2013; de Chassey et al., 2014). This study proves for the first time the interaction between IAV NP and host FLNA protein. In addition, the IAV NP-host FLNA interaction was found to be conserved across multiple IAV isolates. FLNA and NP co-localization was also observed in the nucleus, perinucleus and cytoplasm therefore, pointing toward the significance and importance of further investigating its implication in viral replication and pathogenesis.

We also reported that IAV NP attenuates FLNA mRNA and protein expression levels. Since IAV NP translocates into the nucleus of the host cell, it is possible that IAV NP interrupts the transcriptional activity of Filamin A thus, resulting in the decrease in FLNA mRNA and subsequently, protein levels. We also observed that NP is an essential viral protein required to attain FLNA attenuation in an IAV microenvironment. Furthermore, silencing endogenously expressed FLNA in an IAV microenvironment resulted in a significantly higher virus titer while over-expression of FLNA resulted in a dramatic decrease in virus titer. Collectively, our results substantiate FLNA as a host protein with anti-viral potential and suggest that the IAV strategically attenuates FLNA for efficient viral replication and pathogenesis.

c-Jun N-terminal kinases (JNKs) are a family of protein kinases involved in regulating the host stress signaling pathway and reported to be implicated in host gene expression, cell death and senescence regulation (Yarza et al., 2016). JNK pathway activation and subsequently apoptosis induction are a hallmark of IAV infection and are factors essential for efficient IAV replication (Halder et al., 2011; Cannon et al., 2014; Nacken et al., 2014; Mehrbod et al., 2019; Zhang et al., 2019). Previous studies have reported the role of IAV NS1 in JNK stress signaling pathway activation (Nacken et al., 2014). To the best of our knowledge, our findings which indicate the role of NP in the JNK pathway activation and subsequent apoptosis induction is novel. It is not to our surprise that NP was found to play a role in apoptosis induction post-IAV infection as studies performed priorly in our lab suggested p53 activation, attenuation of anti-apoptotic API5 protein and Clusterin-Bax mediated apoptosis induction upon NP transfection (Tripathi et al., 2013; Mayank et al., 2015; Nailwal et al., 2015). FLNA has been reported to interact with TRAF2, a protein necessary for JNK pathway activation. We observed that the presence of NP disrupted FLNA-TRAF2 interaction thus, resulting in increased levels of free TRAF2 protein available to interact with and activate ASK1, subsequently resulting in the JNK pathway activation.

We also showed in this study that downstream apoptosis-related proteins of the JNK signaling pathway, such as caspase 3, c-Jun, Bad, Bax, and p53, were activated in the presence of IAV NP thus, resulting in the observed increase in cell death. JNKs have been reported to phosphorylate c-Jun, a transcription factor implicated in apoptosis induction, thus resulting in its activation (Liu and Lin, 2005). The tumor protein 53, or p53 in short, is a transcriptional target of activated c-Jun. JNKs have also been reported to phosphorylate p53, resulting in its activation. Bad, Bax, and caspase 3 are downstream proapoptotic effectors known to be activated upon p53 phosphorylation (Supplementary Figure 15; Cregan et al., 1999; Chen et al., 2006).

In addition, we also found that the apoptosis induction, as observed by the Phospho-p53, Phospho-c-Jun, Phospho-Bad, Bax, and activated-caspase 3 levels, via the JNK pathway activation in an IAV microenvironment was significantly increased in FLNA depleted cells. FLNA overexpression, on the other hand, resulted in decreased JNK pathway activation and subsequently, decreased levels of cell death activation. We hypothesize that the protective effect of FLNA against the IAV is through the suppression of the JNK stress signaling pathway thus, creating an inhospitable environment for IAV replication. This ideology is supported by the decrease in virus titer/replication observed post-FLNA overexpression. On the other hand, FLNA silencing may have created a conducive environment for IAV replication by enhancing JNK pathway activation and apoptosis induction.

In general, we have uncovered in this study that the disruption of FLNA-TRAF2 interaction by IAV NP and the attenuation of FLNA post-IAV infection are some of the strategies used by the IAV to activate the JNK stress-signaling pathway for achieving efficient viral replication. The interaction of NP with FLNA to modulate JNK activation and subsequently, apoptosis-related protein activation is a direct proof of the significance of FLNA in the IAV replication cycle hitherto unknown. Due to the conserved nature of the FLNA-NP interaction, we prove FLNA as a novel and attractive target for the development of anti-influenza drug.

## Data Availability Statement

All datasets presented in this study are included in the article/Supplementary Material.

## Author Contributions

SL, SS, and JP contributed to the conception or design of the work. AS, JB, OS, and MR contributed in data collection. AS, MR, and SL contributed in data analysis and interpretation. AS and SL contributed to drafting the article. AS, SS, SL, and VC contributed to the critical revision of the article. All authors contributed to the article and approved the submitted version.

## Conflict of Interest

The authors declare that the research was conducted in the absence of any commercial or financial relationships that could be construed as a potential conflict of interest.

## Abbreviations

A549: adenocarcinomic human alveolar basal epithelial cells
Co-IP: Co-immunoprecipitation
FLNA: Filamin A
h.p.i: hours post-infection
HEK293: human embryonic kidney cells
*HK*: A/Hong Kong/1/1968 (H3N2) isolate
*HKX31*: A/HKx31 (H3N2) isolate
hrs: hours
IAV: influenza A virus
JNK: c-JunN-terminal kinases
M1: matrix 1 protein
MDCK: Madin-Darby Canine Kidney cells
MOI: multiplicity of infection
NP: nucleoprotein
NS1: non-structural 1 protein
NT/CTRL siRNA: non-targeting control siRNA
*PR8*: A/Puerto Rico/8/34 (H1N1) isolate
S.D.: standard deviation
UI/I: uninfected/infected
*WSN*: A/WSN/1933 (H1N1) isolate.

## References

[B1] Batra J. Tripathi S. Kumar A. Katz J. M. Cox N. J. Lal R. B. (2016). Human heat shock protein 40 (HSP40/DnaJB1) promotes influenza A virus replication by assisting nuclear import of viral ribonucleoproteins. *Sci. Rep.* 6:19063. 10.1038/srep19063 PMC4707480 26750153

[B2] Burns L. H. Wang H. Y. (2017). Altered filamin A enables amyloid beta-induced tau hyperphosphorylation and neuroinflammation in Alzheimer’s disease. *Neurol. Neuroimmunol.* 4 263–271. 10.20517/2347-8659.2017.50 PMC8294116 34295950

[B3] Campos L. S. Rodriguez Y. I. Leopoldino A. M. Hait N. C. Bergami P. L. Castro M. G. (2016). Filamin A expression negatively regulates sphingosine-1-phosphate-induced NF-κB activation in melanoma cells by inhibition of Akt signaling. *Mol. Cell. Biol.* 36 320–329. 26552704 10.1128/MCB.00554-15 PMC4719300

[B4] Cannon G. Callahan M. A. Gronemus J. Q. Lowy R. J. (2014). Early activation of MAP kinases by influenza A virus X-31 in murine macrophage cell lines. *PLoS One* 9:e105385. 10.1371/journal.pone.0105385 25166426 PMC4148262

[B5] Chen L. H. Hsu C. Y. Weng C. F. (2006). Involvement of P53 and Bax/Bad triggering apoptosis in thioacetamide-induced hepatic epithelial cells. *World J. Gastroentrol.* 12 5175–5181. 10.3748/wjg.v12.i32.5175 PMC4088015 16937528

[B6] Cooper J. Liu L. Woodruff E. A. Taylor H. E. Goodwin J. S. Aquila R. T. D. (2011). Filamin A protein interacts with human immunodeficiency virus type 1 Gag protein and contributes to productive particle assembly. *J. Biol. Chem.* 286 28498–28510. 10.1074/jbc.m111.239053 21705339 PMC3151092

[B7] Cregan S. P. Maclaurin J. G. Craig C. G. Robertson G. S. Nicholson D. W. Park D. S. (1999). Bax-dependent caspase-3 activation is a key determinant in p53-induced apoptosis in neurons. *J. Neurosci.* 19 7860–7869. 10.1523/jneurosci.19-18-07860.1999 10479688 PMC6782440

[B8] de Chassey B. Meyniel-Schicklin L. Vonderscher J. André P. (2014). Virus-host interactomics: new insights and opportunities for antiviral drug discovery. *Genome Med.* 6:115. 10.1186/s13073-014-0115-1 PMC4295275 25593595

[B9] Deng W. Lopez-Camacho C. Tang J. Y. Mendoza-Villanueva D. Maya-Mendoza A. Jackson D. A. (2012). Cytoskeletal protein filamin A is a nucleolar protein that suppresses ribosomal RNA gene transcription. *Proc. Natl. Acad. Sci. U.S.A.* 109 1524–1529. 10.1073/pnas.1107879109 22307607 PMC3277164

[B10] Diaz A. Allerson M. Culhane M. Sreevarsan S. (2013). Antigenic drift of H1N1 influenza A virus in pigs with and without passive immunity. *Influenza Other Resp.* 7 52–60. 10.1111/irv.12190 24224820 PMC4942991

[B11] Dyer M. D. Murali T. M. Sobral B. W. (2008). The landscape of human proteins interacting with viruses and other pathogens. *PLoS Pathog.* 4:e32. 10.1371/journal.pone.0100032 18282095 PMC2242834

[B12] Falsey A. R. Walsh E. E. (2006). Viral pneumonia in older adults. *Clin. Infect. Dis.* 42 518–524. 10.1086/499955 16421796 PMC7107847

[B13] Feng Y. Walsh C. A. (2004). The many faces of filamin: a versatile molecular scaffold for cell motility and signaling. *Nat. Cell Biol.* 6 1034–1038. 10.1038/ncb1104-1034 15516996

[B14] Ghosh S. Ahrens W. A. Phatak S. U. Hwang S. Schrum L. W. Bonkovsky H. L. (2011). Association of filamin A and vimentin with hepatitis C virus proteins in infected human hepatocytes. *J. Viral Hepat.* 18 e568–e577. 21914078 10.1111/j.1365-2893.2011.01487.x

[B15] Halder U. C. Bagchi P. Chattopadhyay S. Dutta D. Chawla-Sarkar M. (2011). Cell death regulation during influenza A virus infection by matrix (M1) protein: a model of viral control over the cellular survival pathway. *Cell Death Dis.* 2:e197. 10.1038/cddis.2011.75 21881599 PMC3186897

[B16] He W. Wang W. Han H. Wang L. Zhang G. Gao B. (2013). Molecular basis of live-attenuated influenza virus. *PLoS One* 8:e60413 10.1371/journal.pone.060413 PMC3608614 23555969

[B17] Jiménez-Baranda S. Gómez-Moutón C. Rojas A. Martínez-Prats L. Mira E. Ana L. R. (2007). Filamin-A regulates actin-dependent clustering of HIV receptors. *Nat. Cell Biol.* 9 838–846. 10.1038/ncb1610 17572668

[B18] Kälin S. Amstutz B. Gastaldelli M. Wolfrum N. Boucke K. Havenga M. (2010). Macropinocytotic uptake and infection of human epithelial cells with species B2 adenovirus type 35. *J. Virol.* 84 5336–5350. 10.1128/jvi.02494-09 20237079 PMC2863792

[B19] Komarova A. V. Combredet C. Meyniel-Schicklin L. Chapelle M. Caignard G. Camadro J. M. (2011). Proteomic analysis of virus-host interactions in an infectious context using recombinant viruses. *Mol. Cell. Proteom.* 10:M110.007443. 10.1074/mcp.M110.007443 PMC3237069 21911578

[B20] Külshammer E. Uhlirova M. (2012). The actin cross-linker Filamin/Cheerio mediates tumor malignancy downstream of JNK signaling. *J. Cell Sci.* 126 927–938. 10.1242/jcs.114462 23239028

[B21] Leonardi A. Ellinger-Ziegelbauer H. Franzoso G. Brown K. Siebenlist U. (2000). Physical and functional interaction of filamin (actin-binding protein-280) and tumor necrosis factor receptor-associated factor 2. *J. Biol. Chem.* 275 271–278. 10.1074/jbc.275.1.271 10617615

[B22] Li J. Yu M. Zheng W. Liu W. (2015). Nucleocytoplasmic shuttling of influenza A Virus proteins. *Viruses* 7 2668–2683. 10.3390/v7052668 26008706 PMC4452925

[B23] Linero F. N. Sepúlveda C. S. Giovannoni F. Castilla V. Garcia C. C. Scolaro L. A. (2012). Host cell factors as antiviral targets in Arenavirus infection. *Viruses* 4 1569–1591. 10.3390/v4091569 23170173 PMC3499820

[B24] Liu J. Lin A. (2005). Role of JNK activation in apoptosis: a double-edged sword. *Cell Res.* 15 36–42. 10.1038/sj.cr.7290262 15686625

[B25] Liu Q. Zhou Y. Yang Z. (2016). The cytokine storm of severe influenza and development of immunomodulatory therapy. *Cell. Mol. Immunol.* 13 3–10. 10.1038/cmi.2015.74 26189369 PMC4711683

[B26] Malathi K. Siddiqui M. A. Sayal S. Maji M. Ezelle H. J. Zeng C. (2014). RNase L interacts with Filamin A to regulate actin dynamics and barrier function for viral entry. *mBio* 5:e02012-14. 10.1128/mBio.02012-14 PMC4217177 25352621

[B27] Mayanagi T. Morita T. Hayashi K. Fukumoto K. Sobue K. (2008). Glucocorticoid receptor-mediated expression of caldesmon regulates cell migration via the reorganization of the actin cytoskeleton. *J. Biol. Chem.* 283 31183–31196. 10.1074/jbc.m801606200 18772142 PMC2662183

[B28] Mayank A. K. Sharma S. Nailwal H. Lal S. K. (2015). Nucleoprotein of influenza A virus negatively impacts antiapoptotic protein API5 to enhance E2F1-dependent apoptosis and virus replication. *Cell Death Dis.* 6:e2018. 10.1038/cddis.2015.360 26673663 PMC4720893

[B29] Mehrbod P. Ande S. R. Alizadeh J. Rahimizadeh S. Shariati A. Malek H. (2019). The roles of apoptosis, autophagy and unfolded protein response in arbovirus, influenza virus, and HIV infections. *Virulence* 10 376–413. 10.1080/21505594.2019.1605803 30966844 PMC6527025

[B30] Munier S. Rolland T. Diot C. Jacob Y. Naffakh N. (2013). Exploration of binary virus-host interactions using an infectious protein complementation assay. *Mol. Cell. Proteom.* 12 2845–2855. 10.1074/mcp.m113.028688 23816991 PMC3790295

[B31] Nacken W. Anhlan D. Hrincius E. R. Mostafa A. Wolff T. Sadewasser A. (2014). Activation of c-jun N-terminal kinase upon influenza A virus (IAV) infection is independent of pathogen-related receptors but dependent on amino acid sequence variations of IAV NS1. *J. Virol.* 88 8843–8852. 10.1128/jvi.00424-14 24872593 PMC4136289

[B32] Nailwal H. Sharma S. Mayank A. K. Lal S. K. (2015). The nucleoprotein of influenza A virus induces p53 signaling and apoptosis via attenuation of host ubiquitin ligase RNF43. *Cell Death Dis.* 6:e1768. 10.1038/cddis.2015.131 25996295 PMC4669709

[B33] Nishitoh H. Saitoh M. Mochida Y. Takeda K. Nakano H. Rothe M. (1998). ASK1 is essential for JNK/SAPK activation by TRAF2. *Mol. Cell* 2 389–395. 10.1016/s1097-2765(00)80283-x 9774977

[B34] Nomachi A. Nishita M. Inaba D. Enomoto M. Hamasaki M. Minami Y. (2008). Receptor tyrosine kinase Ror2 mediates Wnt5a-induced polarized cell migration by activating c-Jun N-terminal kinase via actin-binding protein Filamin A. *J. Biol. Chem.* 283 27973–27981. 10.1074/jbc.m802325200 18667433

[B35] Qian X. J. Zhu Y. Z. Zhao P. Qi Z. T. (2016). Entry inhibitors: new advances in HCV treatment. *Emerg. Microb. Infect.* 5:e3. 10.1038/emi.2016.3 PMC4735057 26733381

[B36] Samji T. (2009). Influenza A: understanding the viral life cycle. *Yale J. Biol. Med.* 82 153–159. 20027280 PMC2794490

[B37] Savoy R. M. Ghosh P. M. (2013). The dual role of filamin A in cancer: can’t live with (too much of) it, can’t live without it. *Endocr. Relat. Cancer* 20 R341–R356. 24108109 10.1530/ERC-13-0364 PMC4376317

[B38] Shafren D. R. Bates R. C. Agrez M. V. Herd R. L. Bruns G. F. Barry R. D. (1995). Coxsackieviruses B1, B3, and B5 use decay accelerating factor as a receptor for cell attachment. *J. Virol.* 69 3873–3877. 10.1128/jvi.69.6.3873-3877.1995 7538177 PMC189108

[B39] Shaikh F. Y. Utley T. J. Craven R. E. Rogers M. C. Lapierre L. A. Goldenring J. R. (2012). Respiratory syncytial virus assembles into structured filamentous virion particles independently of host cytoskeleton and related proteins. *PLoS One* 7:e40826. 10.1371/journal.pone.0040826 22808269 PMC3396619

[B40] Sharma S. Mayank A. K. Nailwal H. Tripathi S. Patel J. P. Bowzard J. B. (2014). Influenza A viral nucleoprotein interacts with cytoskeleton scaffolding protein a-actinin-4 for viral replication. *FEBS J.* 281 2899–2914. 10.1111/febs.12828 24802111 PMC7164065

[B41] Shimizu K. (2000). Mechanisms of antigenic variation in Influenza virus. *Nihon Rinsho.* 58 2199–2205. 11225304

[B42] Soleimani G. Akbarpour M. (2011). Clinical presentation of novel Influenza A (H1N1) in Hospitalized Children. *Iran J. Pediatr.* 21 215–219. 23056790 PMC3446154

[B43] Stossel T. P. Condeelis J. Cooley L. Hartwig J. H. Noegel A. Schleicher M. (2001). Filamins as integrators of cell mechanics and signaling. *Nat. Rev. Mol. Cell Biol.* 2 138–145. 10.1038/35052082 11252955

[B44] Taubenberger J. K. Morens D. M. (2009). Pandemic influenza - including a risk assessment of H5N1. *Rev. Sci.* 28 187–202. 10.20506/rst.28.1.1879 19618626 PMC2720801

[B45] Thorne L. Arias A. Goodfellow I. (2016). Advances toward a Norovirus antiviral: from classical inhibitors to lethal mutagenesis. *J. Infect.* 213 S27–S31. 10.1093/infdis/jiv280 PMC4704654 26744429

[B46] Tripathi S. Batra J. Cao W. Sharma K. Patel J. R. Ranjan P. (2013). Influenza A virus nucleoprotein induces apoptosis in human airway epithelial cells: implications of a novel interaction between nucleoprotein and host protein Clusterin. *Cell Death Dis.* 4:e562. 10.1038/cddis.2013.89 23538443 PMC3615740

[B47] Wang J. Zhao S. Wei Y. Zhou Y. Shore P. Deng W. (2016). Cytoskeletal filamin A differentially modulates RNA polymerase III gene transcription in transformed cell lines. *J. Biol. Chem.* 291 25239–25246. 10.1074/jbc.m116.735886 27738102 PMC5122789

[B48] Wang Q. Zheng W. Wang Z. Yang J. Hussein S. Tang J. (2015). Filamin-A increases the stability and plasma membrane expression of polycystin-2. *PLoS One* 10:e0123018. 10.1371/journal.pone.0123018 25861040 PMC4393133

[B49] Yang Z. Yang W. Lu M. Li Z. Qiao X. Sun B. (2018). Role of the c-Jun N-terminal kinase signaling pathway in the activation of trypsinogen in rat pancreatic acinar cells. *Int. J. Mol. Med.* 41 1119–1126. 29207022 10.3892/ijmm.2017.3266

[B50] Yarza R. Vela S. Solas M. Ramirez M. J. (2016). c-Jun N-terminal kinase (JNK) signaling as therapeutic target for Alzheimer’s disease. *Front. Microbiol.* 6:321. 10.3389/fphar.2015.00321 26793112 PMC4709475

[B51] Yue J. Huhn S. Shen Z. (2013). Complex roles of filamin-A mediated cytoskeleton network in cancer progression. *Cell Biosci.* 3:7. 10.1186/2045-3701-3-7 23388158 PMC3573937

[B52] Zhang J. Ruan T. Sheng T. Wang J. Sun J. Wang J. (2019). Role of c-Jun terminal kinase (JNK) activation in influenza A virus-induced autophagy and replication. *Virology* 526 1–12. 10.1016/j.virol.2018.09.020 30316042 PMC6424123

