# Supplementary material for: Influenza A Virus Nucleoprotein Activates the JNK Stress-Signaling Pathway for Viral Replication by Sequestering Host Filamin A Protein
Source: Front Microbiol. 2020 Sep 25;11:581867. doi: 10.3389/fmicb.2020.581867 (PMC7546217; doi:10.3389/fmicb.2020.581867)
Supplement: Supplementary file 1 [file Data_Sheet_1.docx]

**
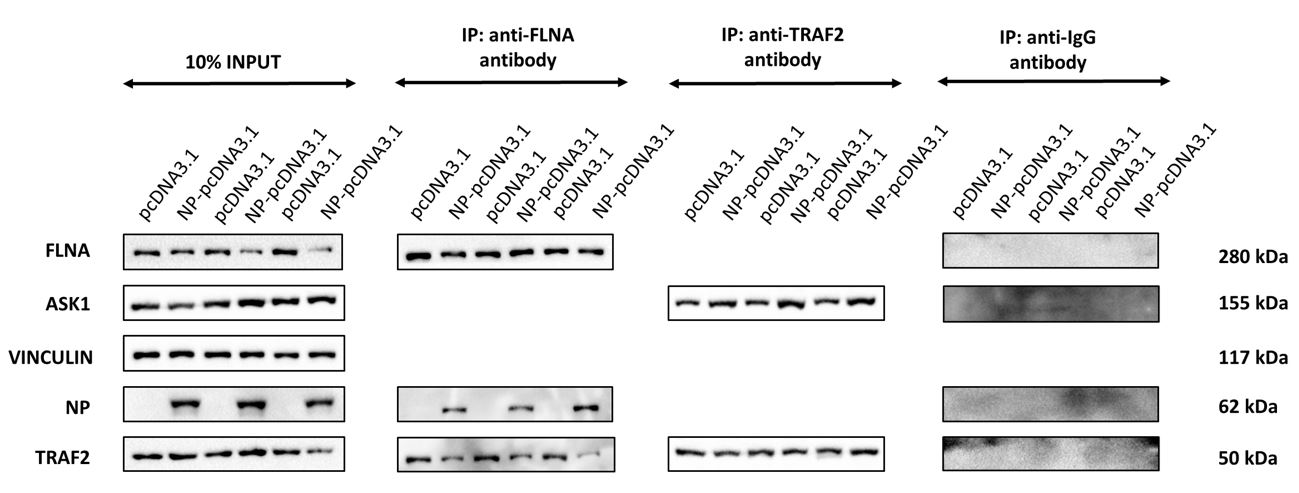
**

**Figure S1.** IAV NP *(PR8)* interrupts FLNA-TRAF2 interaction, allowing for increased TRAF2-ASK1 interaction. HEK293 cells were transfected with either pcDNA3.1 empty control vector or NP(*PR8*) plasmid. The cells were harvested 24 hrs post-transfection and IP was setup using mouse anti-FLNA, mouse anti-TRAF2 and mouse anti-IgG antibody. The eluate from IP was subjected to SDS-PAGE and Western blotting. Blots were developed by ECL for FLNA, vinculin (loading control), ASK1, NP and TRAF2 protein levels (n=3).


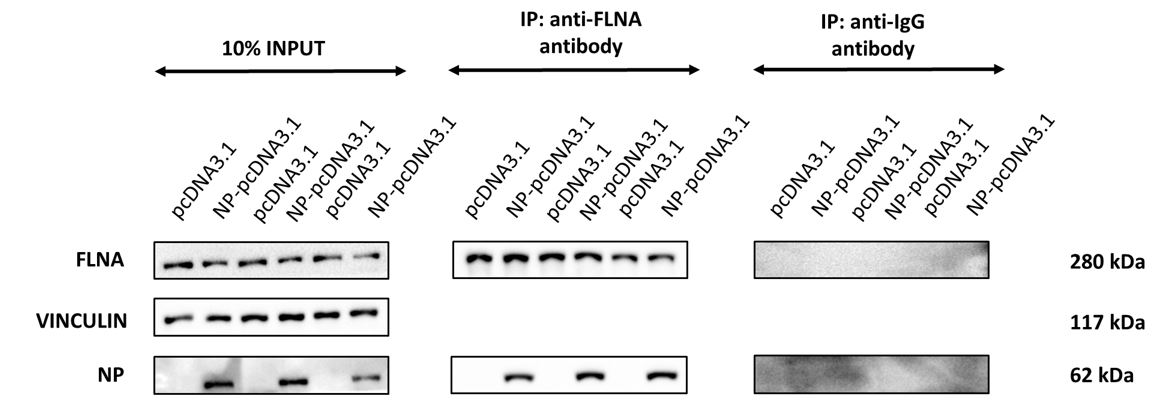


**Figure S2.** Host FLNA interacts with IAV NP (*WSN*). HEK293 cells were transfected with either pcDNA3.1 empty control vector or NP(*WSN*) plasmid. The cells were harvested 24 hrs post-transfection and IP was setup using mouse anti-FLNA and mouse anti-IgG antibody. The eluate from IP was subjected to SDS-PAGE and Western blotting. Blots were developed by ECL for FLNA, vinculin (loading control) and NP protein levels (n=3).


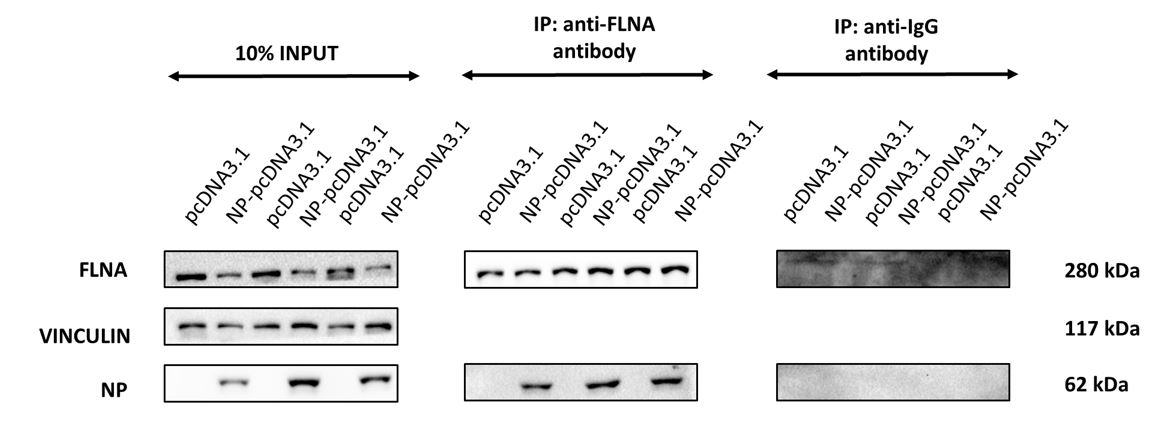


**Figure S3.** Host FLNA interacts with IAV NP (*HK*). HEK293 cells were transfected with either pcDNA3.1 empty control vector or NP(*HK*) plasmid. The cells were harvested 24 hrs post-transfection and IP was setup using mouse anti-FLNA and mouse anti-IgG antibody. The eluate from IP was subjected to SDS-PAGE and Western blotting. Blots were developed by ECL for FLNA, vinculin (loading control) and NP protein levels (n=3).


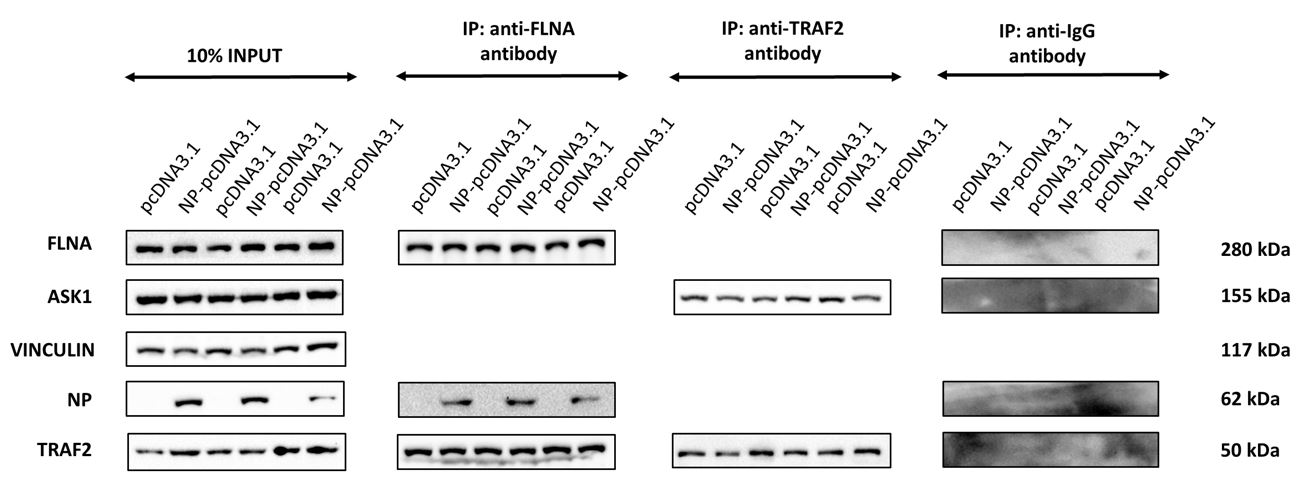


**Figure S4.** IAV NP (*PR8*) interrupts does not interrupt the FLNA-TRAF2 interaction and TRAF2-ASK1 interacting during early timepoints. HEK293 cells were transfected with either pcDNA3.1 empty control vector or NP(*PR8*) plasmid. The cells were harvested 8 hrs post-transfection and IP was setup using mouse anti-FLNA, mouse anti-TRAF2 and mouse anti-IgG antibody. The eluate from IP was subjected to SDS-PAGE and Western blotting. Blots were developed by ECL for FLNA, vinculin (loading control), ASK1, NP and TRAF2 protein levels (n=3).


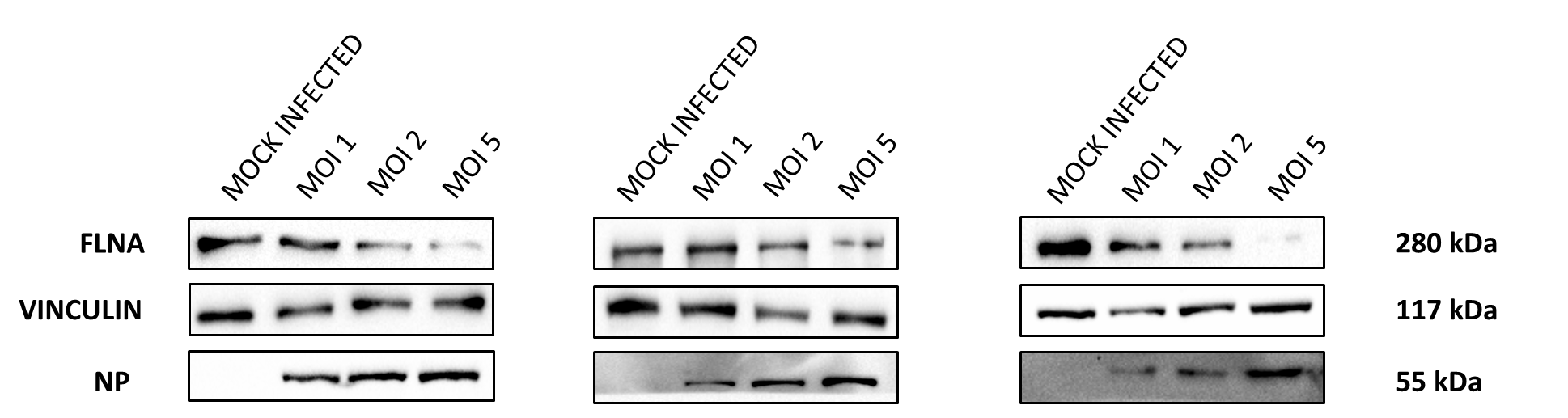


**Figure S5.** IAV infection significantly attenuates FLNA protein levels. A549 cells were infected with IAV *PR8* in a dose-dependent manner. 24 h.p.i the cells were harvested with RIPA buffer and 30µg of protein lysate were loaded onto an SDS-PAGE gel followed by Western blotting. Blots were developed by ECL for FLNA, vinculin (loading control), and NP protein levels (n=3).


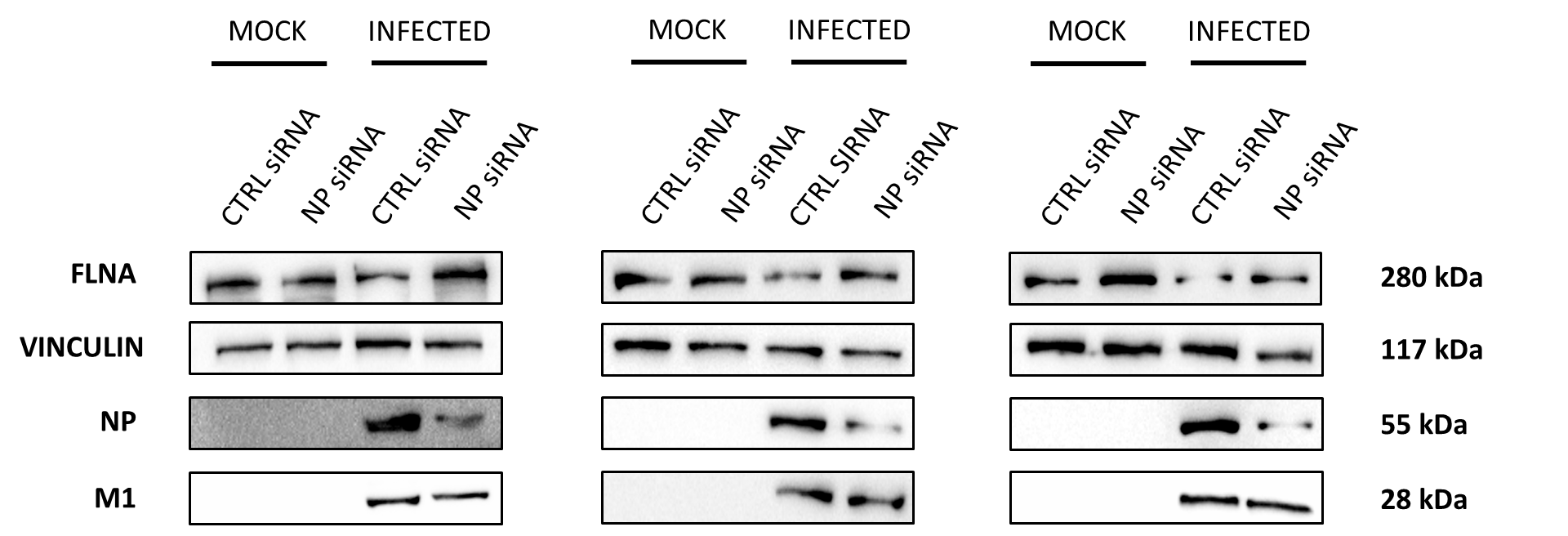


**Figure S6.** The NP of IAV is essential to attain FLNA attenuation post-IAV infection. (A) A549 cells were transfected with control or NP siRNA (100nM) for 24 hrs followed by infection (MOI 5). 24 h.p.i, the cells were harvested with RIPA buffer and 30µg of protein lysate were loaded onto a SDS-PAGE gel followed by Western blotting. Blots were developed by ECL for FLNA, NP, M1, and vinculin (loading control) protein levels (n=3).

**Table S1.** Plaque count and PFU/mL post- control siRNA and FLNA siRNA transfection (200nM) in an IAV *PR8* microenvironment.

|  | **CTRL siRNA** | **FLNA siRNA** |  |  | **CTRL siRNA** | **FLNA siRNA** |
| --- | --- | --- | --- | --- | --- | --- |
| **10^-1^ Plaque Count** | 80 | 160 |  | **PFU/mL** | 5333.33 | 10666.67 |
|  | 79 | 147 |  |  | 5266.67 | 9800.00 |
|  | 79 | 139 |  |  | 5266.67 | 9266.67 |
| **AVERAGE** | 79.33 | 148.67 |  | **AVERAGE** | 5288.89 | 9911.11 |
| **PERCENTAGE** | 100.00 | 187.39 |  | **STD DEV.** | 38.49 | 706.58 |

**Table S2.** Plaque count and PFU/mL post- pEGFP-N1 and FLNA-GFP plasmid transfection (3µg) in an IAV *PR8* microenvironment.

|  | **pEGFP-N1** | **FLNA-GFP** |  |  | **pEGFP-N1** | **FLNA-GFP** |
| --- | --- | --- | --- | --- | --- | --- |
| **10^-1^ Plaque Count** | 150 | 57 |  | **PFU/ML** | 10000.00 | 3800.00 |
|  | 127 | 58 |  |  | 8466.67 | 3866.67 |
|  | 119 | 65 |  |  | 7933.33 | 4333.33 |
| **AVERAGE** | 132 | 60 |  | **AVERAGE** | 8800.00 | 4000.00 |
| **PERCENTAGE** | 100.00 | 45.45 |  | **STD DEV.** | 1072.90 | 290.59 |


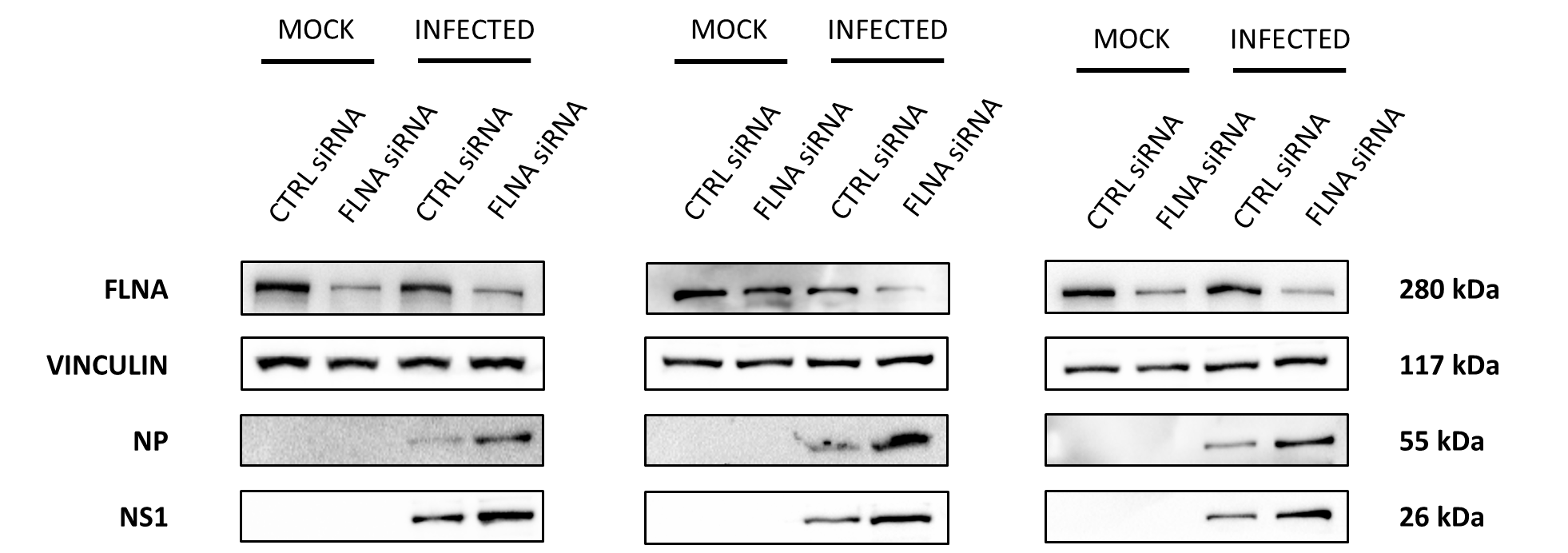


**Figure S7.** NP levels significantly increased post-FLNA silencing in an IAV microenvironment. A549 cells were transfected with FLNA (200nM) or control (CTRL) siRNA (200nM) for 24 hrs followed by IAV *PR8* infection (MOI 5). 24 h.p.i the cells were harvested with RIPA buffer and 30µg of protein lysate were loaded onto an SDS-PAGE gel followed by Western blotting. Blots were developed by ECL for FLNA protein, vinculin protein (loading control), NP and NS1 (n=3).


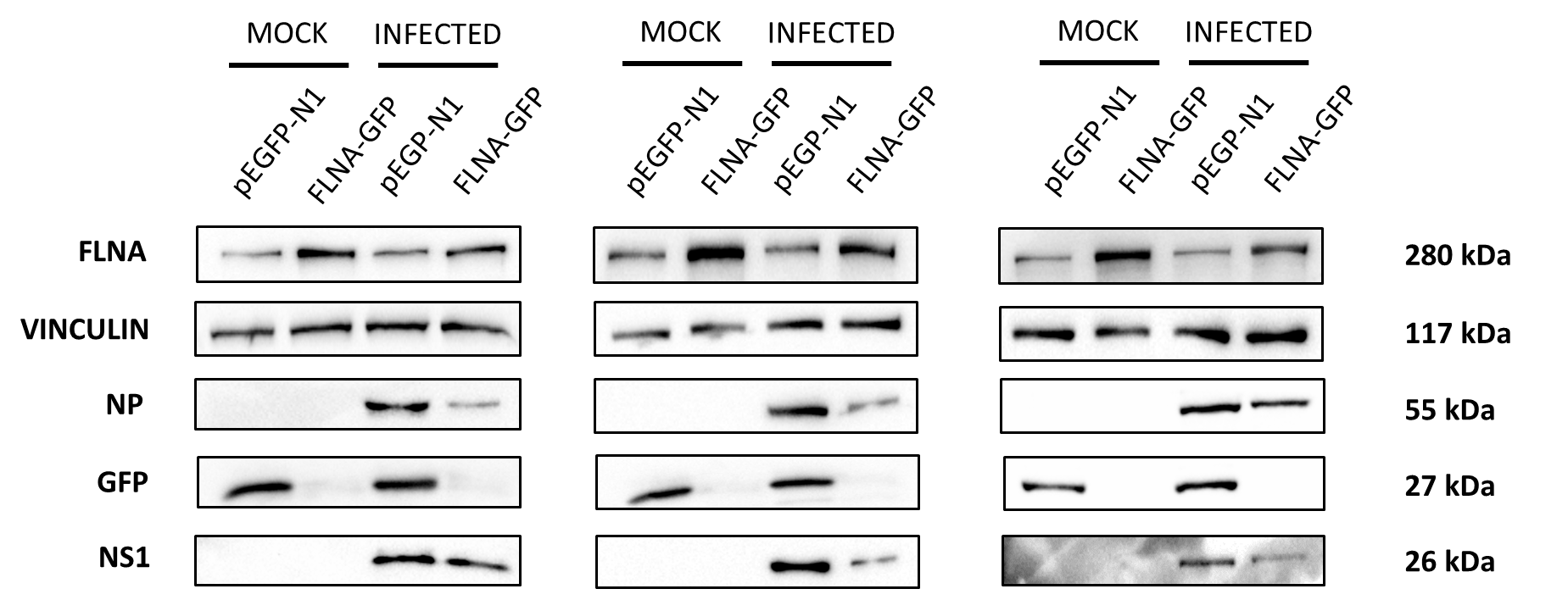


**Figure S8.** NP levels significantly attenuated post-FLNA over-expression. HEK293 cells were transfected with pEGFP-N1 or FLNA-GFP plasmid (3µg) for 24 hrs followed by IAV *PR8* infection (MOI 5). 24 h.p.i the cells were harvested with RIPA buffer and 30µg of protein lysate were loaded onto an SDS-PAGE gel followed by Western blotting. Blots were developed by ECL for FLNA protein, vinculin protein (loading control), NP, GFP and NS1 (n=3).


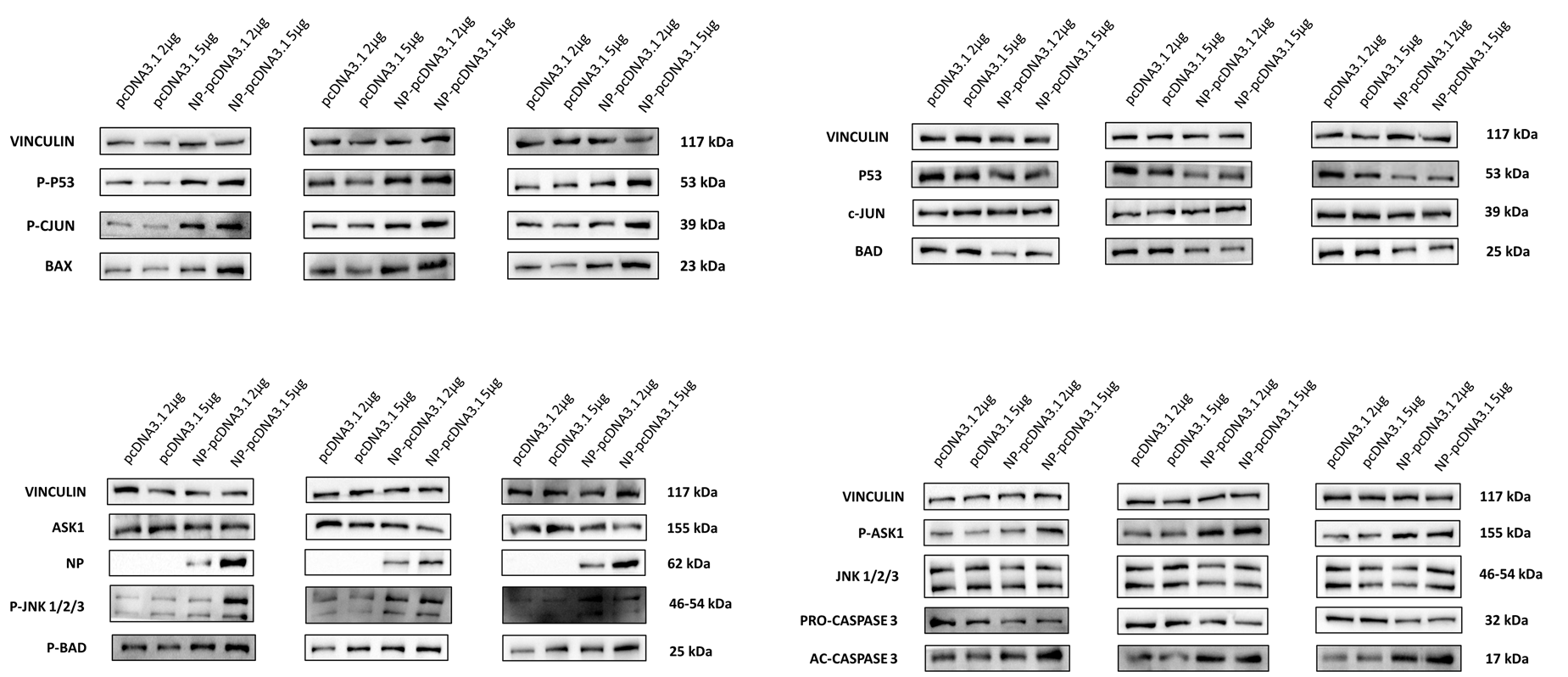


**Figure S9.** IAV NP induces apoptosis in mammalian cells. HEK293 cells were transfected with either pcDNA3.1 empty control plasmid or NP *PR8* plasmid in a dose-dependent manner. The cells were harvested with RIPA buffer 24 hrs post-transfection and 30µg of protein lysate was loaded onto an SDS-PAGE gel followed by Western blotting. Blots were developed by ECL for FLNA, vinculin (loading control), NP and JNK stress signaling pathway-associated markers. Representative blots are shown from one independent experiment (n=3).


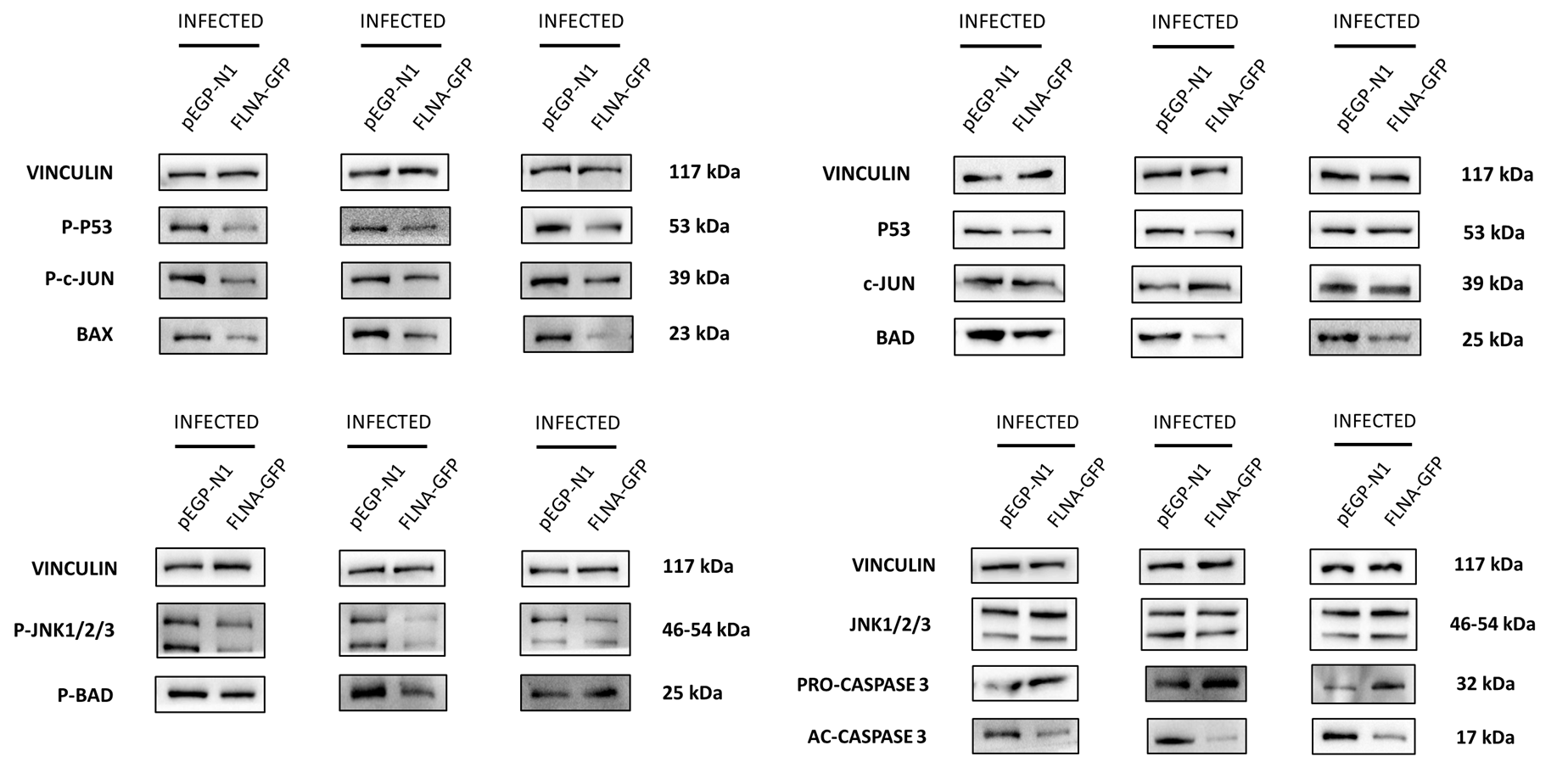


**Figure S10.** FLNA over-expression resulted in decreased levels of JNK stress signaling pathway post-IAV infection. A549 cells were transfected with either control pEGFP-N1 plasmid (3 µg) or FLNA-GFP plasmid (3 µg) followed by IAV *PR8* infection (MOI=5) 24 hrs post-transfection. The cells were harvested with RIPA buffer 24 h.p.i and 30µg of protein lysate was loaded onto an SDS-PAGE gel followed by Western blotting. Blots were developed by ECL for JNK stress signaling pathway-associated markers. Representative blots are shown from one independent experiment (n=3).


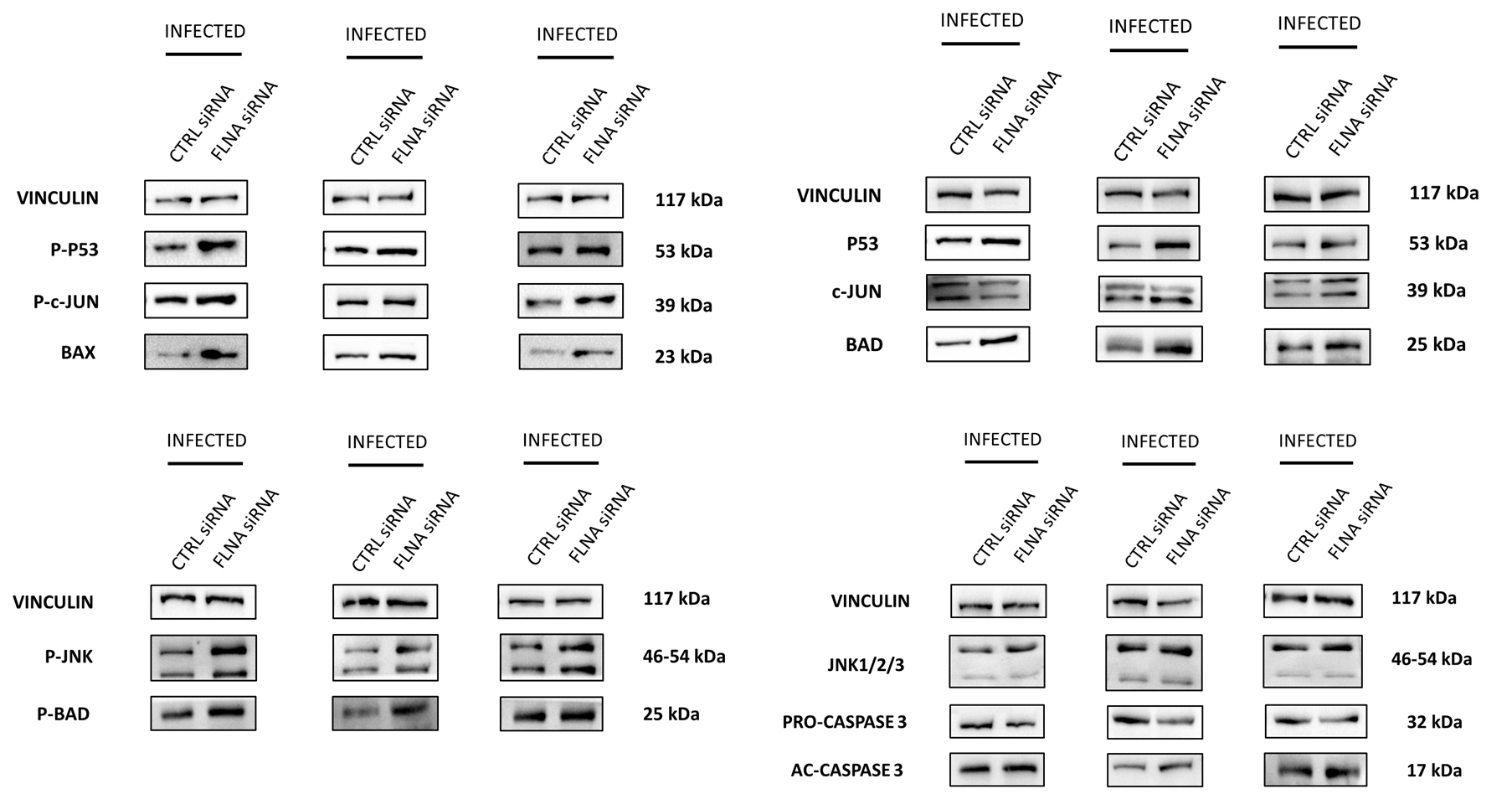


**Figure S11**. FLNA silencing resulted in increased levels of JNK stress signaling pathway post-IAV infection. A549 cells were transfected with either non-targeting control siRNA (200nM) or FLNA siRNA (200nM) followed by IAV *PR8* infection (MOI=5) 24 hrs post-transfection. The cells were harvested with RIPA buffer 24 h.p.i and 30µg of protein lysate was loaded onto an SDS-PAGE gel followed by Western blotting. Blots were developed by ECL for JNK stress signaling pathway-associated markers. Representative blots are shown from one independent experiment (n=3).


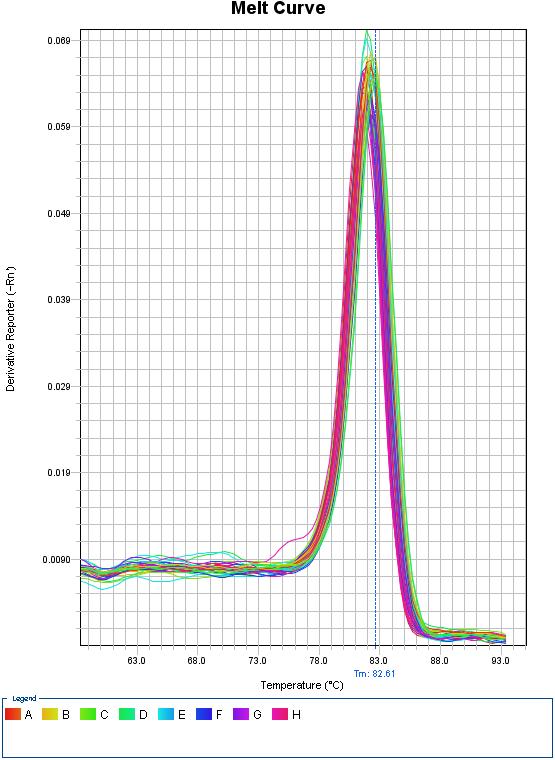


**A**


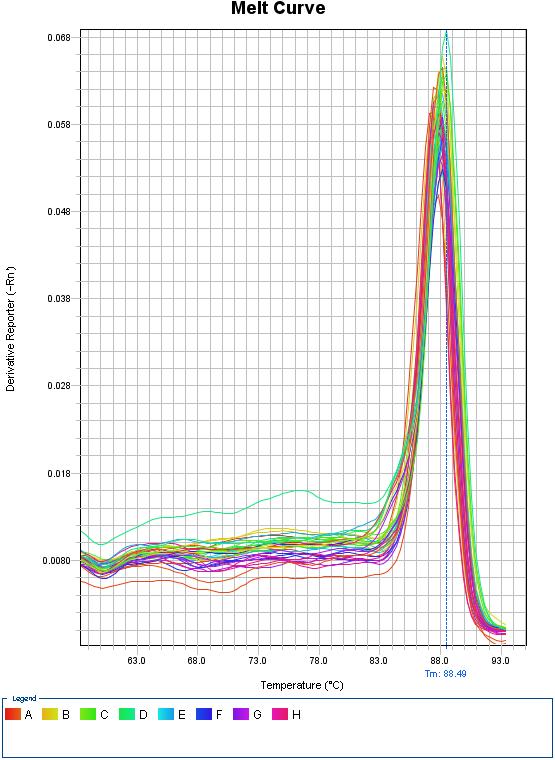


**B**


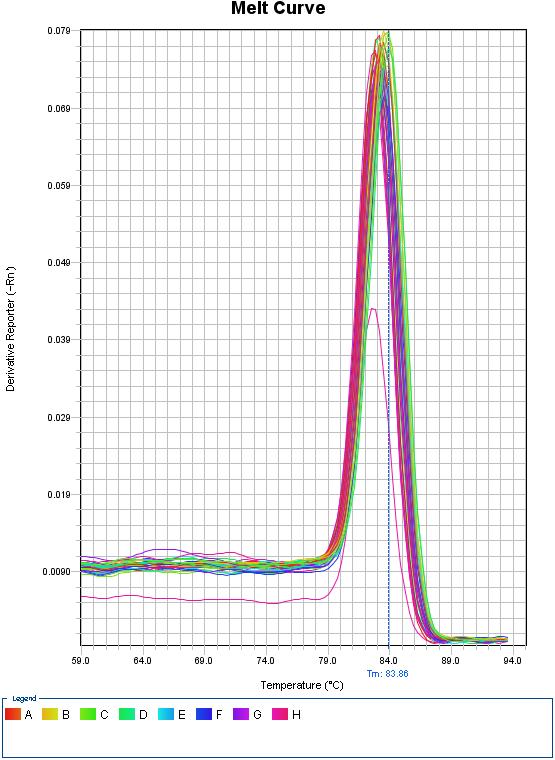


**C**


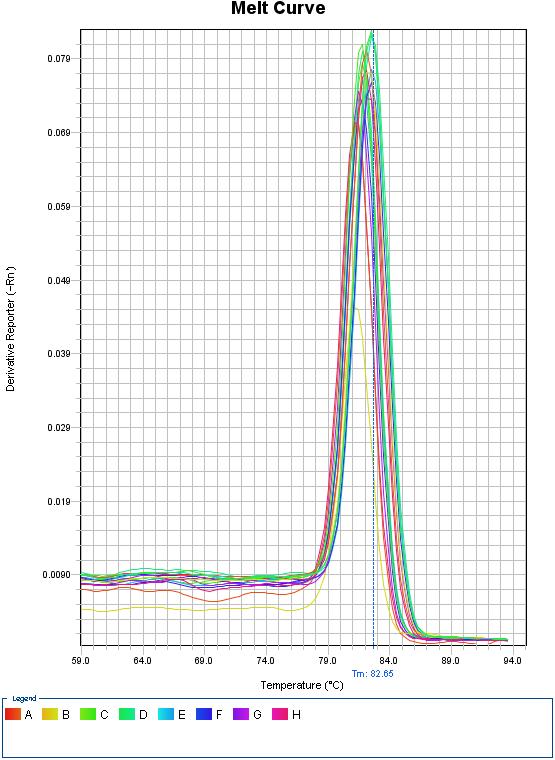


**D**

**Figure S12.** Representative melt curve for (A) β-ACTIN (B) FLNA (C) GAPDH (D) NP.


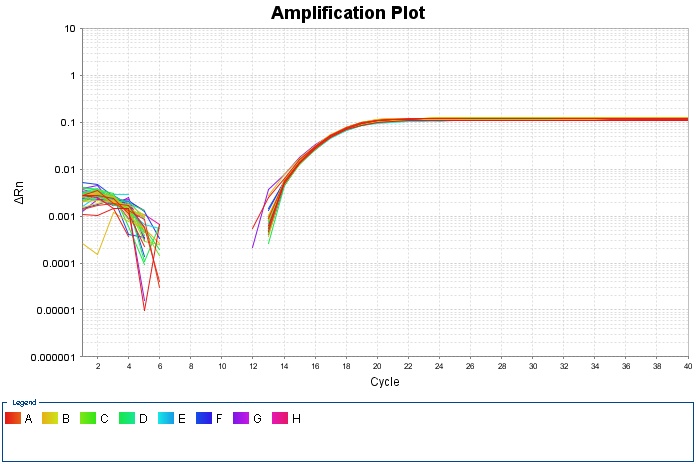


**A**


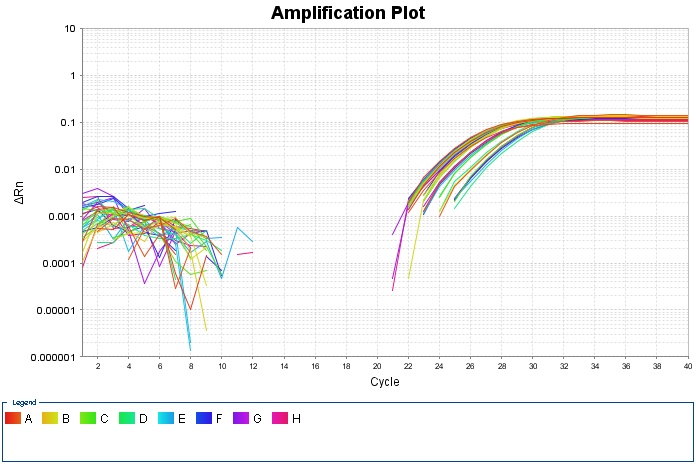


**B**


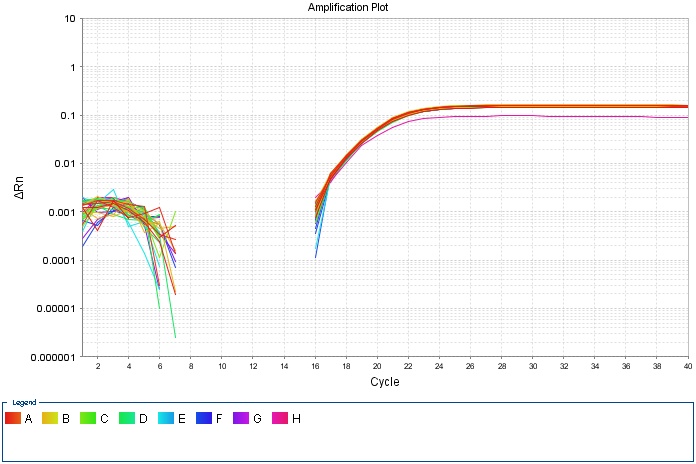


**C**


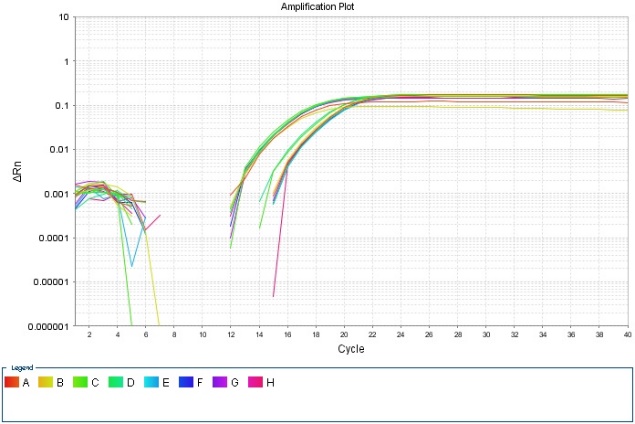


**D**

**Figure S13.** Representative amplification curve for (A) β-ACTIN (B) FLNA (C) GAPDH (D) NP.


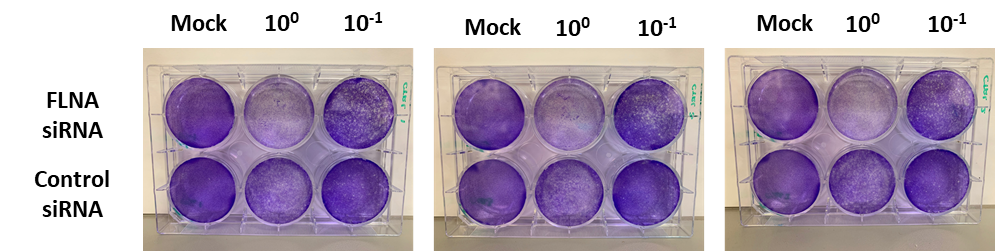


**A**

**B**


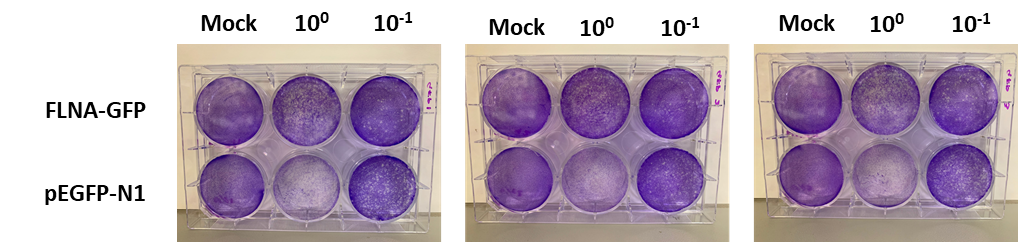


**Figure S14.** FLNA silencing significantly increases viral replication while FLNA over-expression significantly impairs viral replication. (A) Supernatant from siRNA treated and IAV *PR8* infected A549 cells were collected 24 h.p.i to perform plaque assay on MDCK cells. (B) Supernatant from plasmid treated and IAV *PR8* infected HEK293 cells were collected 24 h.p.i to perform plaque assay on MDCK cells.


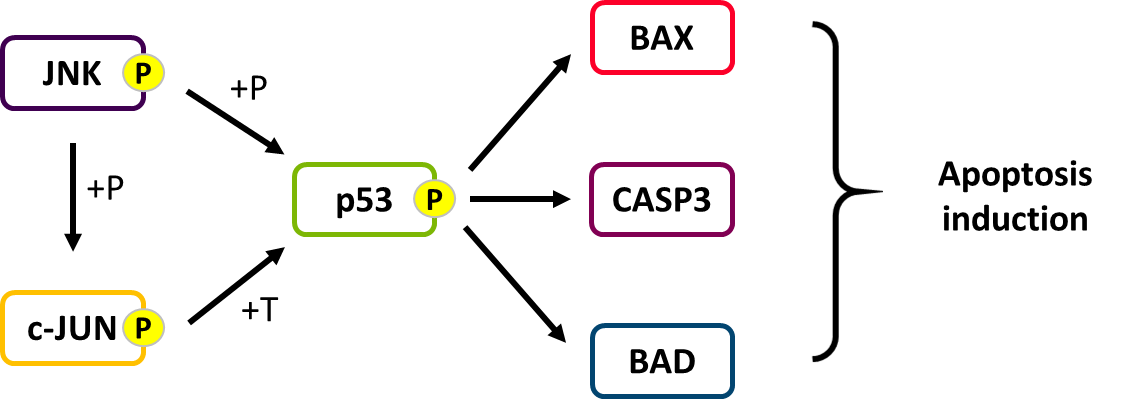


**Figure S15.** The JNK stress signaling pathway model (+P = phosphorylation; +T = transcription).


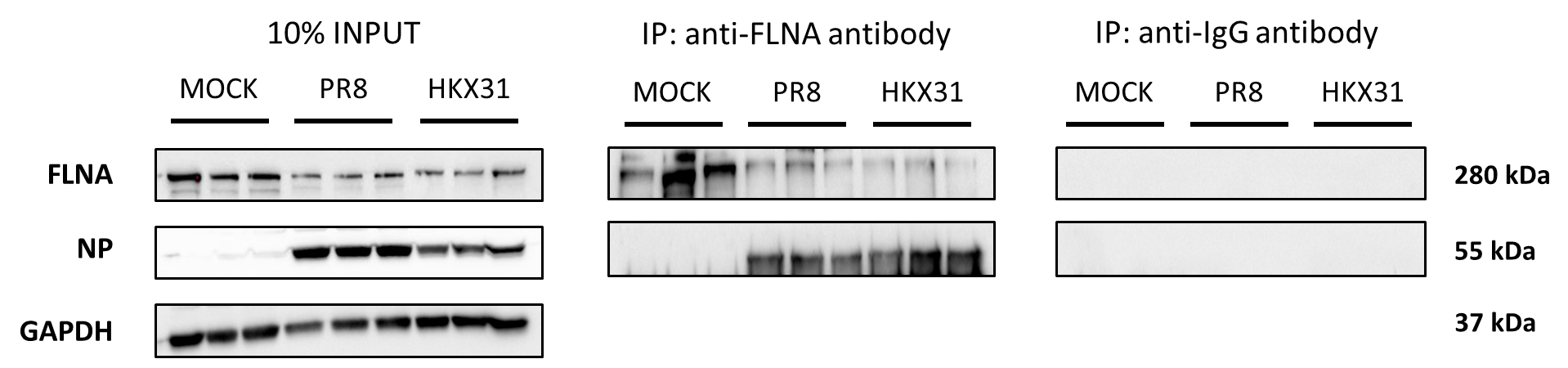


**Figure S16.** A549 cells were infected with IAV *PR8* (MOI=5) and *HKX31* (MOI=1) (n=3). IP was setup using mouse anti-FLNA antibody and anti-IgG mouse antibody and NP was detected in the anti-FLNA antibody IP eluate by Western blotting using mouse anti-NP antibody.
